# Multiple Germline Events Contribute to Cancer Development in Patients with Li-Fraumeni Syndrome

**DOI:** 10.1158/2767-9764.CRC-22-0402

**Published:** 2023-05-01

**Authors:** Vallijah Subasri, Nicholas Light, Nisha Kanwar, Jack Brzezinski, Ping Luo, Jordan R. Hansford, Elizabeth Cairney, Carol Portwine, Christine Elser, Jonathan L. Finlay, Kim E. Nichols, Noa Alon, Ledia Brunga, Jo Anson, Wendy Kohlmann, Kelvin C. de Andrade, Payal P. Khincha, Sharon A. Savage, Joshua D. Schiffman, Rosanna Weksberg, Trevor J. Pugh, Anita Villani, Adam Shlien, Anna Goldenberg, David Malkin

**Affiliations:** 1 Department of Medical Biophysics, University of Toronto, Toronto, Ontario, Canada.; 2 Program in Genetics and Genome Biology, The Hospital for Sick Children, Toronto, Ontario, Canada.; 3 Vector Institute, Toronto, Ontario, Canada.; 4 Institute of Medical Science, University of Toronto, Toronto, Ontario, Canada.; 5 Department of Paediatric Laboratory Medicine, The Hospital for Sick Children, Toronto, Ontario, Canada.; 6 Division of Haematology/Oncology, The Hospital for Sick Children, Department of Paediatrics, University of Toronto, Toronto, Ontario, Canada.; 7 Princess Margaret Cancer Center, University Health Network, Toronto, Ontario, Canada.; 8 Children's Cancer Centre, Royal Children's Hospital, Melbourne, Victoria, Australia.; 9 Murdoch Children's Research Institute, Parkville, Victoria, Australia.; 10 Department of Pediatrics, University of Melbourne, Melbourne, Australia.; 11 Michael Rice Cancer Centre, Women's and Children's Hospital, North Adelaide, South Australia, Australia.; 12 South Australia Health and Medical Research Institute, Adelaide, South Australia, Australia.; 13 South Australia Immunogenomics Cancer Institute, University of Adelaide, Adelaide, Australia.; 14 Department of Paediatrics, London Health Sciences Centre and Western University, London, Ontario, Canada.; 15 Department of Paediatrics, McMaster University, Hamilton, Ontario, Canada.; 16 Department of Medical Oncology, Princess Margaret Hospital and Mount Sinai Hospital, Toronto, Ontario, Canada.; 17 Faculty of Medicine, University of Toronto, Toronto, Ontario, Canada.; 18 Neuro-Oncology Program, Nationwide Children's Hospital and The Ohio State University, Columbus, Ohio.; 19 Department of Oncology, St Jude Children's Research Hospital, Memphis, Tennessee.; 20 Huntsman Cancer Institute, University of Utah, Salt Lake City, Utah.; 21 Clinical Genetics Branch, Division of Cancer Epidemiology and Genetics, NCI, Bethesda, Maryland.; 22 Department of Pediatrics, University of Utah, Salt Lake City, Utah.; 23 PEEL Therapeutics, Inc., Salt Lake City, Utah.; 24 Ontario Institute for Cancer Research, Toronto, Ontario, Canada.; 25 CIFAR: Child and Brain Development, Toronto, Ontario, Canada.; 26 Department of Computer Science, University of Toronto, Toronto, Ontario, Canada.

## Abstract

Li-Fraumeni syndrome (LFS) is an autosomal dominant cancer-predisposition disorder. Approximately 70% of individuals who fit the clinical definition of LFS harbor a pathogenic germline variant in the *TP53* tumor suppressor gene. However, the remaining 30% of patients lack a *TP53* variant and even among variant *TP53* carriers*,* approximately 20% remain cancer-free. Understanding the variable cancer penetrance and phenotypic variability in LFS is critical to developing rational approaches to accurate, early tumor detection and risk-reduction strategies. We leveraged family-based whole-genome sequencing and DNA methylation to evaluate the germline genomes of a large, multi-institutional cohort of patients with LFS (*n* = 396) with variant (*n* = 374) or wildtype *TP53* (*n* = 22). We identified alternative cancer-associated genetic aberrations in 8/14 wildtype *TP53* carriers who developed cancer. Among variant *TP53* carriers, 19/49 who developed cancer harbored a pathogenic variant in another cancer gene. Modifier variants in the WNT signaling pathway were associated with decreased cancer incidence. Furthermore, we leveraged the noncoding genome and methylome to identify inherited epimutations in genes including *ASXL1*, *ETV6*, and *LEF1* that confer increased cancer risk. Using these epimutations, we built a machine learning model that can predict cancer risk in patients with LFS with an area under the receiver operator characteristic curve (AUROC) of 0.725 (0.633–0.810).

**Significance:**

Our study clarifies the genomic basis for the phenotypic variability in LFS and highlights the immense benefits of expanding genetic and epigenetic testing of patients with LFS beyond *TP53*. More broadly, it necessitates the dissociation of hereditary cancer syndromes as single gene disorders and emphasizes the importance of understanding these diseases in a holistic manner as opposed to through the lens of a single gene.

## Introduction

Li-Fraumeni syndrome (LFS; OMIM #151623) is a highly penetrant cancer-predisposition disorder associated with pathogenic germline variants in the *TP53* tumor suppressor gene (1). Families affected with LFS are susceptible to a diverse spectrum of neoplasms including, but not limited to, bone and soft-tissue sarcomas, premenopausal breast cancers, carcinomas of the adrenal cortex, leukemias, and various brain tumors (2, 3).

The classical LFS criteria define a proband as having developed a sarcoma before the age of 45 years, with at least one first-degree relative with any cancer before 45 years and an additional first- or second-degree relative with any cancer before 45 years, or a sarcoma at any age (3). The identification of pathogenic germline *TP53* variants in individuals who did not fulfill the strict definition of LFS led to the revised “Chompret” criteria for germline *TP53* testing, which accounts for a broader spectrum of clinical heterogeneity (4). A recent study by Ceyhan-Bersoy and colleagues reinforces the need to consider a molecular definition of the diverse spectrum of LFS phenotypes (5) to improve cancer risk prediction and define the role of other genes in LFS families. Up to 75% of families with classical LFS harbor germline pathogenic/likely pathogenic (P/LP) *TP53* variants. Among P/LP *TP53-*variant carriers, the cumulative lifetime risk of developing cancer is approximately 68% in males and 93% in females (6). However, molecular events that modify cancer risk in *TP53*-variant carriers remain poorly understood (7–10). Furthermore, the identification of genetic alterations that explain the increased cancer risk in *TP53*-wildtype LFS families is critical in guiding the prospective management of these patients.

The incomplete penetrance, despite the presence of pathogenic variants of *TP53* in LFS suggests: (i) the presence of additional genetic and/or epigenetic driver events that contribute to cancer risk in certain individuals; and (ii) resilience mechanisms that explain the absence of cancer in some *TP53*-variant carriers, possibly initiated through alternative variants in compensatory pathways (11). Candidate gene approaches have been used to consider other relevant genetic causes in LFS families (10, 12–20). However, whole-genome DNA sequencing and/or epigenetic analyses have, to date, only been utilized in isolated case reports to explain these gaps in understanding of cancer occurrence (21–24). In this work, we leveraged family-based whole-genome sequencing (WGS) and methylation of DNA procured from peripheral blood leukocytes (PBL) to evaluate the germline genetic and epigenetic landscapes of a large cohort of patients that fit the clinical definitions of LFS (*n* = 396) and who harbor either pathogenic variant (*n* = 374) or wildtype *TP53* (*n* = 22). Overall, our study identifies diagnostic biomarkers, prognostic signatures, actionable therapeutic targets and correlates of clinical heterogeneity that will allow for improved clinical management of patients with LFS.

## Materials and Methods

### WGS Patient Cohort

**FIGURE 1:**
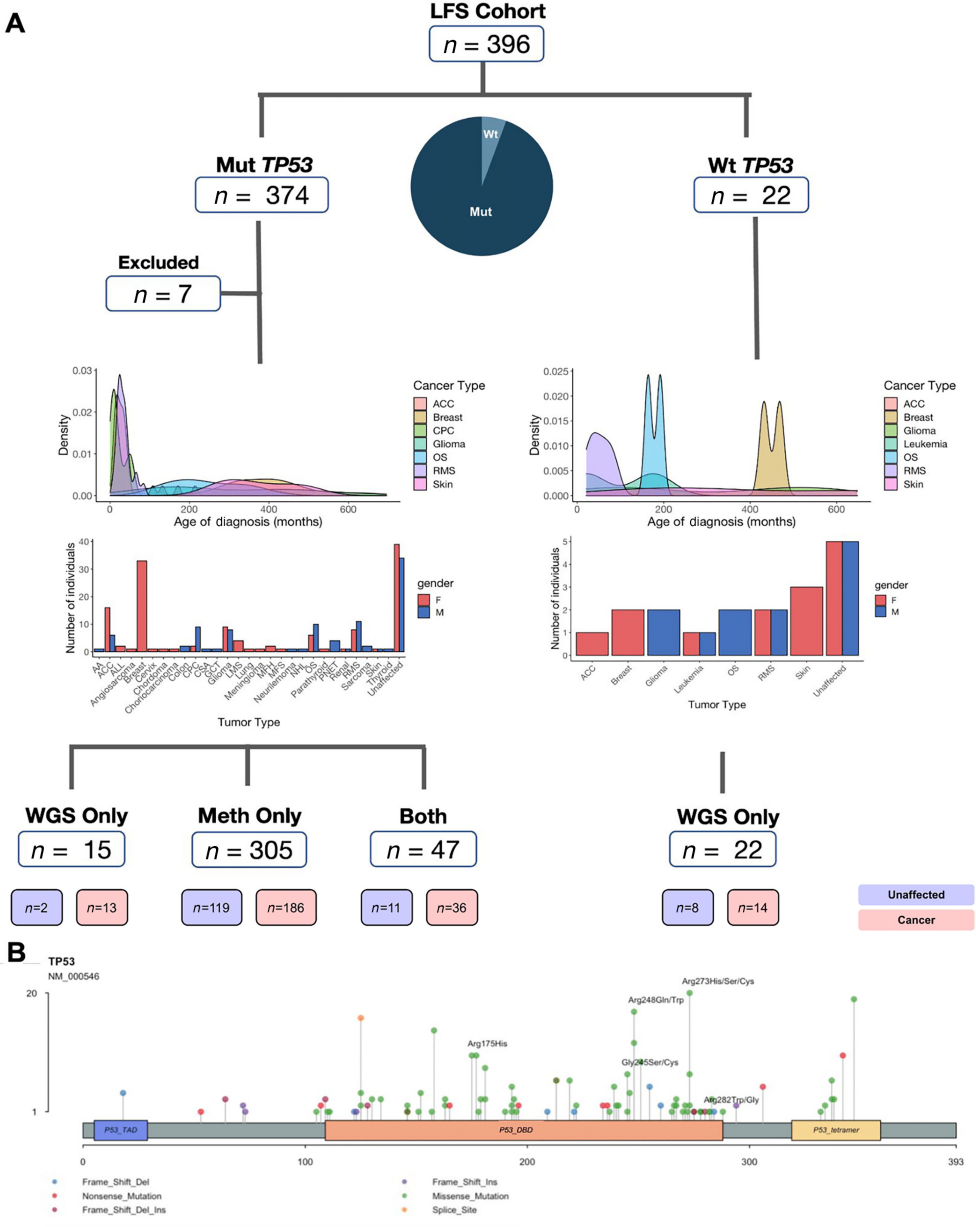
The LFS cohort. **A,** Patient cohort and characteristics (age of first cancer onset, tumor type) by *TP53* status and whether profiling was performed using WGS, methylation or both. AA = adrenal adenoma; ACC = adrenocortical carcinoma; ALL = acute lymphoid leukemia; CPC = choroid plexus carcinoma; CSA = chondrosarcoma; GCT = germ cell tumor; LMS = leiomyosarcoma; MFH = malignant fibrous histiocytoma; MFS = myxofibrosarcoma; NHL = non–Hodgkin lymphoma; OS = osteosarcoma; RMS = rhabdomyosarcoma. **B,** Lollipop plot displaying the location and number of SNVs and indels on the canonical *TP53* transcript: NM_000546, which consists of a TAD, DBD, and tetramerization domain. *TP53* hotspot variants present in our cohort (175, 245, 248, 273, 282) are highlighted.

We performed WGS (RRID:SCR_016385) on 84 LFS family members from 47 families: 22 with wildtype *TP53* and 62 with variant *TP53* (Fig. 1A). Pedigrees for these families were established using information available from clinical notes of probands (Supplementary Figs. S5 and S7). Inclusion criteria for wildtype and variant *TP53* families were determined by resemblance to characteristic patterns of cancer phenotype well documented in literature to be associated with LFS (i) patterns of inheritance (LFS criteria; refs. 1, 4), (ii) cancer type spectrum (adrenal, brain, leukaemia, bone, soft tissue, breast; refs. 6, 25, 26), and (iii) early age of onset that occurs in bimodal cancer-type specific manner (6, 25, 26).

Patterns of inheritance were determined through family history of cancer and characterization of LFS criteria (Supplementary Figs. S5 and S7). The variant *TP53* cohort consists of 49 individuals who developed cancer and 13 individuals who remain cancer-free; 34 were from 13 families with 2–4 individuals sequenced within a given family and the remaining 28 had no family members sequenced. Among the variant *TP53* cohort, two of the families fit the classical LFS criteria, 10 fit the Chompret criteria, and one family fit the incomplete LFS criteria. The wildtype cohort consists of 14 individuals who developed cancer and 8 individuals who are cancer-free, from six families (Fig. 1B). Four of the wildtype *TP53* families fit the Chompret criteria while the remaining two fit the classical LFS criteria. Two to 6 individuals within each *TP53* wildtype family had WGS performed. Moreover, patients in both the variant and wildtype *TP53* cohort develop a similar spectrum of characteristic LFS-associated cancers (e.g., adrenal, brain, soft tissue, bone, blood) and age of cancer onset (Fig. 1A).

### WGS and Processing

A total of 500 ng to 1 μg of genomic DNA was submitted to The Centre for Applied Genomics (TCAG) at The Hospital for Sick Children for genomic library preparation and WGS. DNA samples were quantified using the Qubit High Sensitivity Assay and purity was assessed using the Nanodrop OD 260/280 ratio. Approximately 500–700 ng of DNA was used as input material for library preparation using the Illumina TruSeq PCR-free DNA Library Prep Kit following the manufacturer's recommended protocol. In brief, DNA was fragmented to 400 bp on average using sonication on a Covaris LE220 instrument. Fragmented DNA was end-repaired, A-tailed, and indexed TruSeq Illumina adapters with overhang-T were added to the DNA. Libraries were then validated on a Bioanalyzer DNA High Sensitivity chip to check for size and absence of primer dimers and quantified by qPCR using Kapa Library Quantification Illumina/ABI Prism Kit protocol (KAPA Biosystems). Validated libraries were pooled in equimolar quantities and paired-end sequenced on an Illumina HiSeq X platform following Illumina's recommended protocol to generate paired-end reads of 150 bases in length and an average depth of 40X. FASTQ files were aligned to the hg19 reference genome using BWA-mem v0.78 (ref. 27; RRID:SCR_017619). PCR duplicates were marked with Picard MarkDuplicates v1.1.08 (ref. 28; RRID:SCR_006525), and base recalibration and realignment was performed using GATK v2.8.1 (RRID:SCR_001876; ref. 29).

### Single-nucleotide Variant and Indels

Single-nucleotide variant (SNV) and indel detection was performed using GATK v.4.0.2.1 (RRID:SCR_001876; ref. 29) according to best practices for germline cohort data. All detected variants were further filtered to remove false positives using a set of filters designed for short read sequences as follows: read position 10–90, strandedness 1%–99%, distance to 3′ > 20, homopolymer <5, map quality difference <30, read length difference <25, and MMQS difference<100 (30). Variants with less than five alternate reads detected using bam-readcount were removed.

#### Identification of Pathogenic SNVs and Indels

Population filters were applied to remove common variants found in non-cancer “normal” populations: gnomAD, ExAC and 1kGP, at an allele frequency >0.01 (31). InterVar, an implementation of the ACMG-AMP guidelines (32), was used to classify variants into five categories: pathogenic (P), likely pathogenic (LP), variant of uncertain significance (VUS), likely benign (LB), and benign (B). In addition, all null variants (nonsense, frameshift, canonical ±2 splice sites, initiation codon, single or multiexon deletion) in a gene where loss of function is a known mechanism of disease, that met population filters, were annotated as LP. If the variant was found in all affected members of a family, it was coded as 1 for PP1 and as 0 for BS4. If the variant was not found in any affected members of a family, it was coded as 0 for PP1 and as 1 for BS4. Variants identified in singleton samples were coded as 0 for both PP1 and BS4 (33).

### Structural Variants

#### Structural Variant Discovery

Germline structural variant (SV) discovery was performed using DELLY v0.7.7 (RRID:SCR_004473; ref. 34) and Manta v1.0.3 (35) to discover deletions, duplications, and inversions.

#### Filtering and Annotation of Deletions, Duplications, and Inversions

An ensemble approach was taken, retaining only the deletions, duplications, and inversions called by both DELLY and Manta with a maximum allowed distance of 1 kb between breakpoints. Manta relies on read-pair, split-read, and local-assembly support to call variants, while Delly uses read-pair and split-read support. The following size-specific reciprocal overlap constraints were used for breakpoint consensus between the two tools: breakpoints within 100 bp for SVs ≤10 kbp, breakpoints within 1 kbp for SVs ≥10 kbp and ≤50 kbp and breakpoints within 10 kbp for SVs ≥50 kbp. A high-quality SV set was obtained by applying additional filtering criteria. A panel of SVs occurring as a result of sequencing artefacts was created to remove technical false positives using 72 control germline genomes sequenced on the same HiSeqX platform (150 bp paired end sequencing, minimum of 30X depth coverage). SVs present in ≥3 samples in the panel of normals were removed. Annotation was performed with AnnotSV (36) and subsequently filtered to remove SVs with an AnnotSV score <3 and present in publically available non-cancer “normal” databases: 1000 Genomes Project (RRID:SCR_008801; ref. 37) and gnomAD (RRID:SCR_014964; ref. 38).

### Copy-number Variation

Copy-number variation (CNV) was identified using read depth based CNV detection algorithms: CNVnator (RRID:SCR_010821; ref. 39) and ERDS (40). The intersection of the CNVnator and ERDS calls were subsequently filtered to remove repetitive and low-complexity regions using a predefined list formed by Trost and colleagues (41) This was followed by annotation using ANNOVAR (42).

### Prioritizing Variants Using Tiered Cancer Gene Lists

Although a whole-genome approach was used to detect cancer-associated germline events, four gene lists were compiled to allow for the prioritization of our findings. From highest known cancer predisposition potential to lowest:

**Tier 1: Autosomal dominant cancer predisposition genes** (*n* = 60): 60 autosomal dominant CPG [from Zhang and colleagues (43); Supplementary Table S2].
**Tier 2: Autosomal recessive cancer predisposition genes** (*n* = 29): 29 autosomal recessive CPG [from Zhang and colleagues (43); Supplementary Table S2].
**Tier 3: Cancer genes** (*n* = 489): 58 tumor suppressors, 24 tyrosine kinases and 407 other cancer genes frequently mutated in the somatic context. This list was adapted from Zhang and colleagues (43) with the addition of nine genes that have been added since the publication in 2015, to the COSMIC Cancer Gene Census (Supplementary Table S2; ref. 44).
**Tier 4: All genes** (*n* = 21,380): This list encompasses all the other mapped genes in the genome, not present in the previous tiers.

### Cancer Variant Classification Schema

The modern classification of variants for cancer makes it difficult to distinguish between pathogenic (P)/likely pathogenic (LP), and likely benign (LB)/benign (B) variants because (i) the relationship between a given gene and the cancer developed is not fully understood, and (ii) the functional consequence of a variant in a gene is difficult to comprehend without functional experimentation. As a result, we used a five-tier classification scheme for pathogenicity as follows:

**Class 1:** P/LP genetic variant in a known autosomal dominant, CPG (Tier 1).
**Class 2:** P/LP genetic variant in a known autosomal recessive, CPG (Tier 2).
**Class 3:** P/LP genetic variant in a known cancer gene frequently mutated in the somatic context (Tier 3).
**Class 4:** P/LP genetic variant in novel, candidate cancer gene (Tier 4) included if supported by sufficient evidence in the literature.
**Class 5:** Cancer-segregating VUS in a known cancer gene (Tier 1–3)

### Analysis of WGS Data from 1000 Genomes Project (1kGP)

Genotype data for 2,504 individuals, generated by the 1kGP project in Variant Call Format from version 5 data release were downloaded by ftp (ftp://ftptrace.ncbi.nih.gov/1000genomes/ftp/release/20130502/supporting/vcf_with_sample_level_annotation/; ref. 37). All SNVs, indels, and structural variants were subject to pathogenicity classification as outlined above.

### Analysis of WGS Data from SickKids Cancer Sequencing Program

The SickKids Cancer Sequencing (KiCS) Program is a prospective study of a demographically diverse population of children, adolescents and young adults with refractory, metastatic, relapsed or rare cancers, as well as children with unresolved suspicion for cancer predisposition (45). Whole-genome data from 185 KiCS patients were utilized as a “control” cohort of individuals who developed cancer who lack a germline *TP53* variant. We excluded all patients with a previous diagnosis of a cancer predisposition disorder, more specifically individuals diagnosed with hereditary paraganglioma syndrome and Lynch syndrome. All KiCS FASTQ files were processed through the same pipeline as the LFS FASTQ files. SNVs, indels, and structural variants were also identified and subject to pathogenicity classification using the same methodology as the variants identified in the LFS cohort, as described above.

### Pathway Analysis

The hallmark gene sets from the MSigDB collections were utilized to perform a pathway analysis. Fisher exact test was implemented for each of the 50 pathways, followed by FDR correction (46).

### Survival Analysis Based on P/LP Variants in Cancer Genes

Survival data for 49 patients with WGS were right censored at death or last follow-up (Supplementary Table S1A). Each patient was assigned to a group based on having: (i) at least one class 1–3 variant (*n* = 15), (ii) P/LP variant in the WNT signaling pathway (*n* = 4), and (iii) no class 1–3 or P/LP WNT signaling variant (*n* = 31). Each patient fit the criteria for a single group, with the exception of one patient (Supplementary Fig. S6L – Patient 1355), that harbored a P/LP variant in *AXIN1* (ii) and *XRCC1* (i). Kaplan–Meier survival curves were created using survminer (v0.4.9) and survival (v3.3–1) with *P* values calculated using a log-rank test.

### Methylation Sample Cohort

We profiled DNA methylation of PBLs procured from patients with LFS with a germline *TP53* variant (*n* = 359) from 287 families. A subset of the patients in this cohort overlapped with the WGS cohort (*n* = 47). The number of individuals profiled for methylation per family ranged from 1 to 7 with a median of 1. Our discovery cohort (*n* = 89) consisted of samples collected from previous work (9) composed of individuals with cancer (*n* = 63) and individuals who did not have cancer at the time of analysis (*n* = 26). Methylation analysis for these samples was performed using the Illumina HumanMethylation 450k BeadChip array. Our internal validation cohort (*n* = 124) consisted primarily of patients with a pathogenic germline *TP53* variant from the Hospital for Sick Children who developed cancer (*n* = 78) and those that were cancer-free (*n* = 46). Our external validation cohort (*n* = 146) consisted exclusively of individuals with pathogenic germline *TP53* variants from the NCI who developed cancer (*n* = 88) and those that were unaffected (*n* = 58) (47). Both validation cohorts were profiled using the Illumina EPIC (850k) array. By limiting the samples processed on different array types exclusively to one cohort, we mitigated any biases that may have arisen due to array type.

### Preprocessing and Addressing Confounders in the Methylation Data

Preprocessing and bias correction were performed on the discovery and each validation cohort separately. Raw beta values were corrected for dye-bias using ssNoob (Supplementary Fig. S11; ref. 48). The normalized beta values were then corrected for the batch effects between 96-well plates using ComBat (49). We used Probabilistic Estimation of Expression Residuals (PEER; RRID:SCR_009326)—a factor analysis method that infers hidden determinants and their effects on molecular profiles—to remove broad variance from known confounders (array type, batch, systemic treatment at draw, age of sample collection, gender) as well as 100 hidden (latent) factors (50). Following PCA transformation of each cohort (Supplementary Fig. S12), samples within 3 SDs of the mean of PC1 (μ_PC1_ ± 3σ) and PC2 (μ_PC2_ ± 3σ) were retained. This resulted in the removal of 1, 2, and 4 outliers from the discovery, internal validation, and external validation cohort, respectively, and a modest decrease to the size of our discovery cohort (*n* = 88), internal validation cohort (*n* = 122), and external validation cohort (*n* = 142).

### Discovery of Cancer-associated Secondary Constitutional Epimutation in LFS Patients with a *TP53* Variant

Using the preprocessed methylation data, an Epigenome-Wide Association Study (EWAS) was performed by testing each of the 452,497 methylation probes for their association with cancer status as follows:

- EWAS of the discovery cohort using the Aziz test (ref. 51; methylation ∼ cancer status). *P* values were adjusted using FDR and probes with FDR_discovery_ < 0.1 were considered significant.
- EWAS of the internal and external validation cohorts using the Aziz Test (methylation ∼ cancer status). *P* values were adjusted using FDR and probes with FDR_validation_ < 0.1 in both validation cohorts were considered significant.
- The methylation probes found in the discovery cohort with FDR_discovery_ <0.1 that were validated in both the internal and external validation cohorts with FDR_validation_ < 0.1 resulted in 931 significant EWAS probes.

The patients with both WGS and methylation (*n* = 47) were used to identify cancer-associated secondary constitutional epimutations (CSCE). The premise behind this analysis is that a CSCE at a given probe will reveal underlying genetic cancer predisposition facilitated by epigenetic regulation (52, 53). We focused our analysis on *cis*-CSCE, as we were underpowered for the discovery of *trans*-CSCE due the size of our cohort. A *cis*-CSCE was defined as a EWAS probe significantly associated with a SNP within 10 kb upstream or downstream (*cis*-SNP). This association between a significant EWAS probe and a *cis*-SNP was determined using Spearman correlation. Each SNP was coded additively as 1 (homozygous reference), 3 (heterozygous), or 4 (homozygous alternative). For each EWAS probe, the strongest *cis*-CSCE was retained to represent that probe. Subsequently, FDR correction was performed on all the *cis*-CSCE. Using a FDR cutoff of 0.05 resulted in the discovery of 259 probes with cancer-associated *cis*-CSCEs. We tested all 259 *cis*-CSCE for their association with cancer type using an ANOVA.

### Epimutation Clustering and Survival Analysis

We used Uniform Manifold Approximation and Projection (UMAP) for dimensionality reduction of the 259 *cis*-CSCE with the following parameters: n_neighbors = 15, n_components = 2, metric = euclidean. K-means clustering was used to cluster the UMAP projection; this revealed three distinct clusters of *TP53* variant carriers. Available survival data (*n* = 328) were right censored at death or last follow-up (Supplementary Table S1A). Survival differences were evaluated between the three clusters using Kaplan–Meier survival curve with survminer (v0.4.9) and survival (v3.3-1).

### LFS Cancer Risk Scores

We used methylation at the 259 *cis*-CSCE loci to build a random forest model to predict cancer risk in LFS (caret v6.0.91). To avoid overfitting, the external validation cohort (*n* = 142) was used as the training data, the internal validation cohort (*n* = 122) as the validation data and the discovery cohort (*n* = 88) as the test data. We trained a random forest with repeated 5-fold cross-validation, tuning the number of features considered at each split point (mtry) using grid search. The prediction probabilities from our model can be interpreted as an LFS cancer risk score—an indicator of elevated cancer risk in *TP53* variant carriers. We then used our cancer risk model to predict risk scores on our validation and test sets. The optimal specificity threshold was determined as 0.667 in the discovery cohort using Youden index.

### EWAS Exploring the Effect of Systemic Treatment on Methylation

Systemic treatment information is available for the majority of our internal and external validation cohorts (*n* = 254), consisting of 59 individuals that received systemic treatment prior to sample collection and 195 that received systemic treatment after sample collection or not at all. A total of 40% (78/195) of the individuals that did not receive systemic treatment developed cancer and 60% (117/195) remain cancer-free (Supplementary Table S1A). In addition to correcting for broad variation due to systemic treatment using PEER, we performed an EWAS to identify methylation probes significantly associated with systemic treatment. The Aziz test was used to test the association between each methylation probe and systemic treatment status in the internal and external validation cohorts. *P* values were adjusted using FDR and probes with FDR_validation_ < 0.1 in both validation cohorts were considered significant.

### Feature Enrichment Analysis of Cancer-associated *cis*-CSCE

We performed a feature enrichment analysis of the 259 significant *cis*-CSCE using 10 genomic properties, which included proximity to histone marks, open chromatin and expression levels from blood-derived samples. ChromImpute *P*-value signal tracks (bigwig files) were downloaded from the Roadmap Epigenomics Consortium (https://egg2.wustl.edu/roadmap/data/byFileType/signal/consolidatedImputed/) for the following genomic properties: H3K4me1, H3K4me3, H3K27me3, H3K9me3, H3K27ac, H3K36me3, DNase, H2A.Z, H3K79me2, and RNA-sequencing, in 23 blood-derived samples (E062, E034, E045, E033, E044, E043, E039, E041, E042, E040, E037, E048, E038, E047, E029, E031, E035, E051, E050, E036, E032, E046, E030). A total of 100,000 methylation probes were sampled with replacement to calculate the baseline enrichment level. Average signal values were calculated at the genomic loci of the 100,000 probes for all features in each sample. Average signal values were calculated at the genomic loci of the 259 significant *cis*-CSCE probes for all features in each sample. A paired Wilcoxon test was used to calculate a *P* value between the signal value of the null probes and the *cis*-CSCE probes for each feature. Similarly, effect size was calculated using Cohen distance (d).

### Methylation of *LEF1* in Plasma cell-free DNA of Patients with LFS Compared with Healthy Blood Controls

A total of 173 individuals with a germline *TP53* variant and 28 healthy blood controls (HBC) with wildtype *TP53* were profiled using cell-free methylated DNA immunoprecipitation (cfMeDIP-seq). cfMeDIP-seq samples were processed, aligned, and quantified into nonoverlapping 300 bp bins, as described in detail in another study by our group (54). Using the 300 bp bin at position *chr4:*109056901-109057200, which overlapped the probe *cg03041109,* we compared *LEF1* methylation between HBC and patients with LFS.

### Methylation of *LEF1* in Choroid Plexus Tumors

Genome-wide DNAm profiles were generated using the Illumina HumanMethylation450 BeadChip array for 34 primary samples of choroid plexus tumor (CPT), consisting of 16 choroid plexus carcinomas (CPC), eight with wildtype *TP53* and eight with a germline *TP53* variant*,* and 20 choroid plexus papillomas, 19 with wildtype *TP53* and one with variant *TP53*. All samples were processed as described in detail in our previous work (55, 56). We compared methylation of *LEF1 (cg03041109)* from individuals that developed CPTs, with and without a germline *TP53* variant.

### Methylation of *LEF1* in Wilms Tumors

DNA methylation was collected and processed for 95 individuals from the United Kingdom and 81 from Toronto, Canada, as previously described by our collaborators (57). The UK cohort consisted of 36 normal kidney samples, 22 nephrogenic rest (NR), 32 Wilms tumor (WT) with favorable histology, and five with anaplasia (58). The Toronto cohort consisted of 18 blood (control), 25 kidney samples, 30 WT with favorable histology, and eight with anaplasia. To avoid batch effects, methylation of *LEF1* (cg03041109) was compared between groups, within each cohort.

### Consent

This study was conducted in accordance with the ethical principles of the Declaration of Helsinki and approved by an Institutional Review Board. All LFS (#1000051699) and KiCS (#0019910602) patients were approved for molecular profiling by the SickKids Research Ethics Board. The NCI LFS cohort study was approved by the NCI Institutional Review Board (ClinicalTrials.gov identifier NCT01443468). Written informed consent was signed by all participants or their legal guardian prior to sample collection.

### Data Availability

All WGS and methylation data files are available in the European Genome-Phenome Archive (EGA) under the accession numbers EGAS00001007075 and EGAS00001007061. Analysis scripts can be found at doi:10.5281/zenodo.5563379.

## Results

### Patient Cohort Characteristics

Our international, multi-institutional (*n* = 30) LFS cohort consisted of 396 individuals, 374 with a pathogenic *TP53* variant from 298 families (Supplementary Table S1A), and 22 with wildtype *TP5*3 from six families (Fig. 1A; Supplementary Table S1B). Overall, 18 tumor types are observed among the patients with ages of onset ranging from 0–70.4 years and a mean age at diagnosis of 22.5 years [95% confidence interval: (20.4–24.5); Fig. 1A]. A total of 43% of the family members with cancer developed a tumor by the age of 18. The most prevalent tumor types were breast cancer (*n* = 69), central nervous system tumors (*n* = 40), adrenocortical carcinoma (ACC, *n* = 28), rhabdomyosarcoma (RMS, *n* = 28), and osteosarcoma (*n* = 24). In addition, we profiled 140 cancer-free family members of probands with clinical follow-up information, 8 from wildtype *TP53* families and 132 from variant *TP53* families (Fig. 1A). We performed WGS and/or methylation of DNA from PBL. In total, WGS was performed on 84 patients with wildtype (*n* = 22) and pathogenic *TP53* variants (*n* = 62), and methylation of PBL was performed on 359 patients with LFS with a pathogenic *TP53* variant*.* Given limited sample availability, only a subset (*n* = 47) of the LFS cohort had both WGS and methylation profiling.

### Germline *TP53* Variants

Pathogenic/likely pathogenic (P/LP) germline *TP53* variants in the LFS cohort consisted of SNVs (*n* = 316), deletions (*n* = 41), insertions (*n* = 7), and insertion-deletions (*n* = 11; Fig. 1B). Of the 250 missense variants, 86.4% (*n* = 216) interrupted the DNA-binding domain (DBD), and the remaining 13.6% (*n* = 34) affected the tetramerization domain (TAD; Fig. 1B). Five hotspot residues made up 20.8% of all the variants, consisting of R175 (*n* = 10), R245 (*n* = 8), R248 (*n* = 29), R273 (*n* = 29), and R282 (*n* = 2). The majority of the *TP53* missense variants were cytosine-to-thymine (C>T) transitions (*n* = 126). One patient diagnosed with an anaplastic embryonal rhabdomyosarcoma (ERMS) at age 5 years, harbored a compound heterozygous genotype with two pathogenic *TP53* variants in *trans*: the hotspot R248Q variant (MAF = 0.29) and a pathogenic splicing variant in intron 5 (MAF = 0.22). Another notable patient, harbored an extremely rare, homozygous truncating *TP53* variant and developed two aggressive synchronous primary malignancies (59).

### Pathogenic Germline Genetic Variants in LFS

**FIGURE 2:**
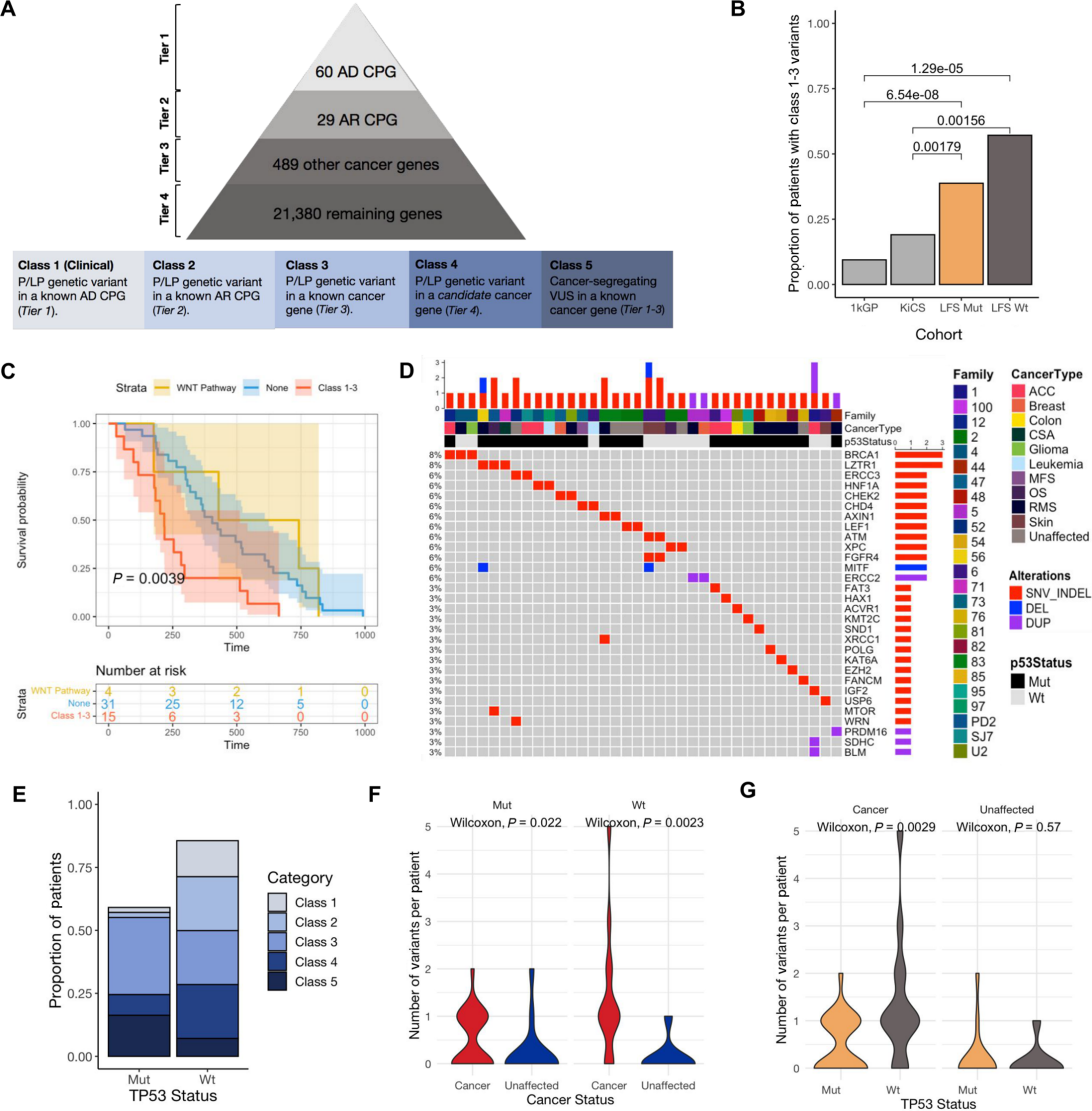
Germline genetic variants in LFS. **A,** Classification scheme for genetic variants identified from WGS. The pyramid (top) shows how genes were prioritized into four tiers from highest known cancer predisposition potential to lowest. All variants were then classified using a *Cancer Variant Classification Schema* (Materials and Methods) into five classes (bottom) based on pathogenicity and whether the gene fell into Tier 1–4. AD: autosomal dominant; AR: autosomal recessive; CPG: cancer predisposition gene. **B,** Frequency of P/LP variants in known cancer genes (class 1–3) in LFS, KiCS and 1kGP. Fisher exact test *P* values for comparisons between the LFS cohorts and KiCS/1kGP shown. **C,** Kaplan–Meier survival curve comparing patients with P/LP variants in the WNT signaling pathway (yellow), patients with at least one class 1–3 variant (red) and patients with neither WNT signaling or class 1–3 variants (blue). **D,** Landscape of genetic alterations in cancer genes (class 1–3) in LFS. Annotated with family, cancer type and *TP53* variant status. Genetic variants are indicated as a deletion (blue), SNV or indel (red) or duplication (purple). **E,** The proportion of individuals in the wildtype and variant *TP53* cohorts with classes 1–5 variants (Materials and Methods). **F,** The number of P/LP variants in cancer genes (class 1–3) in individuals that developed cancer compared with unaffected individuals, stratified by *TP53* status. **G,** The number of P/LP variants (class 1–3) in wildtype versus variant *TP53* carriers, stratified by cancer status. On the basis of the premise that variants in the WNT signaling pathway are associated with decreased cancer incidence in LFS, these variants were removed from the comparisons made in F and G.

WGS of 84 individuals, 62 with variant *TP53* and 22 with wildtype *TP53*, revealed 595,975,507 SNVs/indels and 19,241 SVs. On average, the LFS genomes had 7,261,182 variants per individual (Supplementary Table S1C). All genetic variants were classified into five classes using the *Cancer Variant Classification Schema* as described in the Materials and Methods (Fig. 2A; Supplementary Table S2). Two control cohorts with wildtype *TP53* were used for comparison: (i) whole-exome data from the 1000 Genomes Project (1kGP) consisting of cancer-free individuals (*n* = 2,504) and (ii) whole-genome data from the KiCS Project consisting of individuals with no known cancer predisposition disorder, who developed cancer (*n* = 185). For the subsequent analyses, *TP53* variants were omitted from the LFS cohort as they present an inherent bias to any statistical test comparing variants between cohorts. We compared the frequency of class 1–3 variants (P/LP variants in known cancer genes) between patients with LFS cancer, KiCS (*n* = 185) and 1kGP (*n* = 2,504). The prevalence of class 1–3 variants was 9.42% in 1kGP (Supplementary Table S3) and 19.05% in KiCS (Supplementary Table S4), which was significantly lower than the 39.3% and 57.14% prevalence observed in the cancer-affected *TP53*-variant (Fisher exact test, 1kGP: *P* = 4.98 × 10^−9^; KiCS: *P* = 8.90 × 10^−4^) and *TP53*-wildtype (Fisher exact test, 1kGP: *P* = 1.29 × 10^−5^; KiCS: *P* = 1.56 × 10^−3^) groups (Supplementary Table S5), respectively (Fig. 2B). This suggests that an enrichment of P/LP variants in additional cancer genes among patients with LFS may be contributing to their high rate of malignant transformation. However, the presence of a P/LP variant in a cancer gene (class 1–3) does not appear to contribute to the earlier age of cancer onset that is characteristic of patients with LFS (Supplementary Fig. S1). Furthermore, patients with LFS with at least one class 1–3 variant displayed lower overall survival than individuals lacking a class 1–3 variant (Fig. 2C).

*BRCA1* was the only autosomal dominant CPG (class 1; Table 1) mutated in our cohort. Eight autosomal recessive CPG (class 2; Table 1) and 12 well-characterized cancer genes (class 3) were found to be mutated in the LFS cohort (Fig. 2D). Recurrent P/LP variants were identified in individuals diagnosed with cancer from distinct families, in the following cancer genes: *BRCA1* (*n* = 3)*, LZTR1* (*n* = 3), *HNF1A* (*n* = 2), *CHEK2* (*n* = 2), *MITF* (*n* = 2), and *CHD4* (*n* = 2; Fig. 2D). In addition, 10% (8/84) of patients harbored therapeutically targetable variants, as previously reported in the non-LFS germline context (60), like PARP inhibitors, mTOR inhibitors, EZH2 inhibitors, or tyrosine kinase inhibitors (Supplementary Fig. S2). Pathway analysis of the P/LP SNVs, indels, and SVs across all genes revealed that the p53 pathway (freq = 13%; FDR = 0.19) and the epithelial-to-mesenchymal transition pathway (freq = 13%; FDR = 0.10) were the most frequently mutated pathways across patients with LFS with cancer (Supplementary Fig. S3).

**TABLE 1:** Variants in cancer predisposition genes (class 1–2) in the wildtype and variant *TP53* cohorts, annotated with cancer type, sex, LFS criteria, inheritance status, segregation, variant location, variant function, and zygosity

Patient ID	Family ID	TP53 status	Cancer type (age at diagnosis, months)	Sex	Gene	Class	LFS criteria	Inheritance status	Segregation	Variant	Variant function	Zygosity
3298A	MUT12	Mut	Adrenocortical carcinoma (21)	F	*BRCA1*	1	Unknown	Unknown	Unknown	NM_007294:exon2: c.68_69del:p.E23fs	frameshift deletion	Het
2766	WT4	Wt	Embryonal rhabdomyosarcoma (24)	M	*BRCA1*	1	Classic	Inherited	Cancer segregating	NM_007294:exon10:c. 5266dup:p.Gln1756fs	frameshift deletion	Het
2768	WT4	Wt	Low grade glioma (516)	M	*BRCA1*	1	Classic	Unknown	Cancer segregating	NM_007294:exon10:c. 5266dup:p.Gln1756fs	nonsynonymous SNV	Het
1478	MUT52	Mut	Unaffected	M	*ERCC3*	2	Chompret	Unknown	Unaffected segregating	NM_000122:exon3:c. 325C>T:p.Arg109Ter	nonsynonymous SNV	Het
1478	MUT52	Mut	Unaffected	M	*WRN*	2	Chompret	Unknown	Unaffected segregating	NM_000553:exon24:c. 2856_2857del:p.S952fs	nonsynonymous SNV	Het
3273	MUT76	Mut	Unaffected	M	*FANCM*	2	Classic	Not inherited from Mom	Unaffected segregating	NM_020937:exon11:c. 5791C>T:p.Arg1931Ter	stopgain	Het
SJACT007	MUTSJ7	Mut	Adrenocortical carcinoma	M	*ERCC3*	2	Unknown	Unknown	Unknown	NM_000122:exon8: c.1120_1121insAGCAGT: p.W374delinsX	frameshift deletion	Het
1970	WT2	Wt	Osteosarcoma (164)	M	*XPC*	2	Birch	Inherited	Shared	NM_004628:exon1: c.C55T:p.Q19X	stopgain	Het
1972	WT2	Wt	Unaffected	F	*XPC*	2	Birch	Unknown	Shared	NM_004628:exon1: c.C55T:p.Q19X	stopgain	Het
3021	WT6	Wt	Basal cell carcinoma (648)	F	*ATM*	2	Classic	Unknown	Cancer segregating	NM_000051:exon49: c.T7271G:p.V2424G	splicing	Het
4093	WT6	Wt	Anorectal melanoma (319)	F	*ATM*	2	Classic	Inherited	Cancer segregating	NM_000051:exon49: c.T7271G:p.V2424G	stopgain	Het

### Cancer-associated Germline Genetic Modifiers in LFS Patients with Variant *TP53*

We observed that patients with LFS with a germline *TP53* P/LP variant harbor an additional germline P/LP variant in another cancer gene that may prime them for cancer development (61). Among the patients with variant *TP53* LFS who developed cancer (*n* = 49), 38.8% (19/49) of the patients had a second germline variant in a known cancer predisposition or cancer-associated gene (class 1–3; Fig. 2E; Supplementary Table S5A–S5D). Upon the inclusion of P/LP variants in novel, candidate cancer genes (class 4), we identified a secondary germline variant in 8.2% (4/49) of the patients who developed tumors (Fig. 2E; Supplementary Table S5E and S5F). These additional germline hits were scarce in the cancer-free individuals (Fig. 2F), with the exception of variants in the WNT signaling pathway, which we later discuss as being associated with decreased cancer incidence. With the exclusion of variants in the WNT signaling pathway, variant *TP53* carriers that developed cancer were significantly enriched for class 1–3 variants (Wilcoxon rank-sum test, *P* = 0.022) and class 4 (Wilcoxon rank-sum test, *P* = 0.022) variants in comparison with the cancer-free variant *TP53* carriers (Supplementary Fig. S4). For a subset of the variant *TP53* carriers (*n* = 34), samples from cancer-free family members were available for WGS (Supplementary Fig. S5). For these patients, we performed family-based sequencing analysis, which allowed the identification of disease-segregating variants. Including disease-segregating VUS in known cancer genes (class 5), 12.2% (6/49) of the patients with cancer were found to harbor a secondary germline hit (Supplementary Table S5G).

### Molecular Differences Between LFS Patients with Variant *TP53* Compared to Those Harboring Wildtype *TP53*

Among the patients with LFS who developed cancer (*n* = 63), the wildtype *TP53* patients (*n* = 14) had a greater burden of P/LP variants in CPG (class 1–2; Wilcoxon rank-sum test, *P* = 3.9 × 10^−4^; Supplementary Fig. S4) and cancer genes (class 1–3; Wilcoxon rank-sum test, *P* = 2.9 × 10^−3^) compared with the variant *TP53* cohort (*n* = 49; Fig. 2G). This suggests that in the absence of a strong driver like a pathogenic *TP53* variant, patients with LFS with wildtype *TP53* require a greater burden of P/LP variants in other cancer-associated genes to be primed for cancer development. Compared with the variant *TP53* patients who developed cancer, the wildtype *TP53* patients who developed cancer were enriched for P/LP variants in genes found to be upregulated by reactive oxygen species (ROS; freq = 9%; FDR = 0.11; Supplementary Fig. S6) and genes important for mitotic spindle assembly (freq = 14%; FDR = 0.11; Supplementary Fig. S6). Although no one pathway was significantly enriched in the variant *TP53* cohort over the wildtype cohort, KRAS signaling downregulation approached significance (freq = 15%; FDR = 0.09; Supplementary Fig. S6) as the most frequently mutated pathway in the variant *TP53* cohort, with no P/LP variants identified in the wildtype cohort.

### Identification of Alternative Susceptibility Genes in LFS Patients with Wildtype *TP53*

**FIGURE 3:**
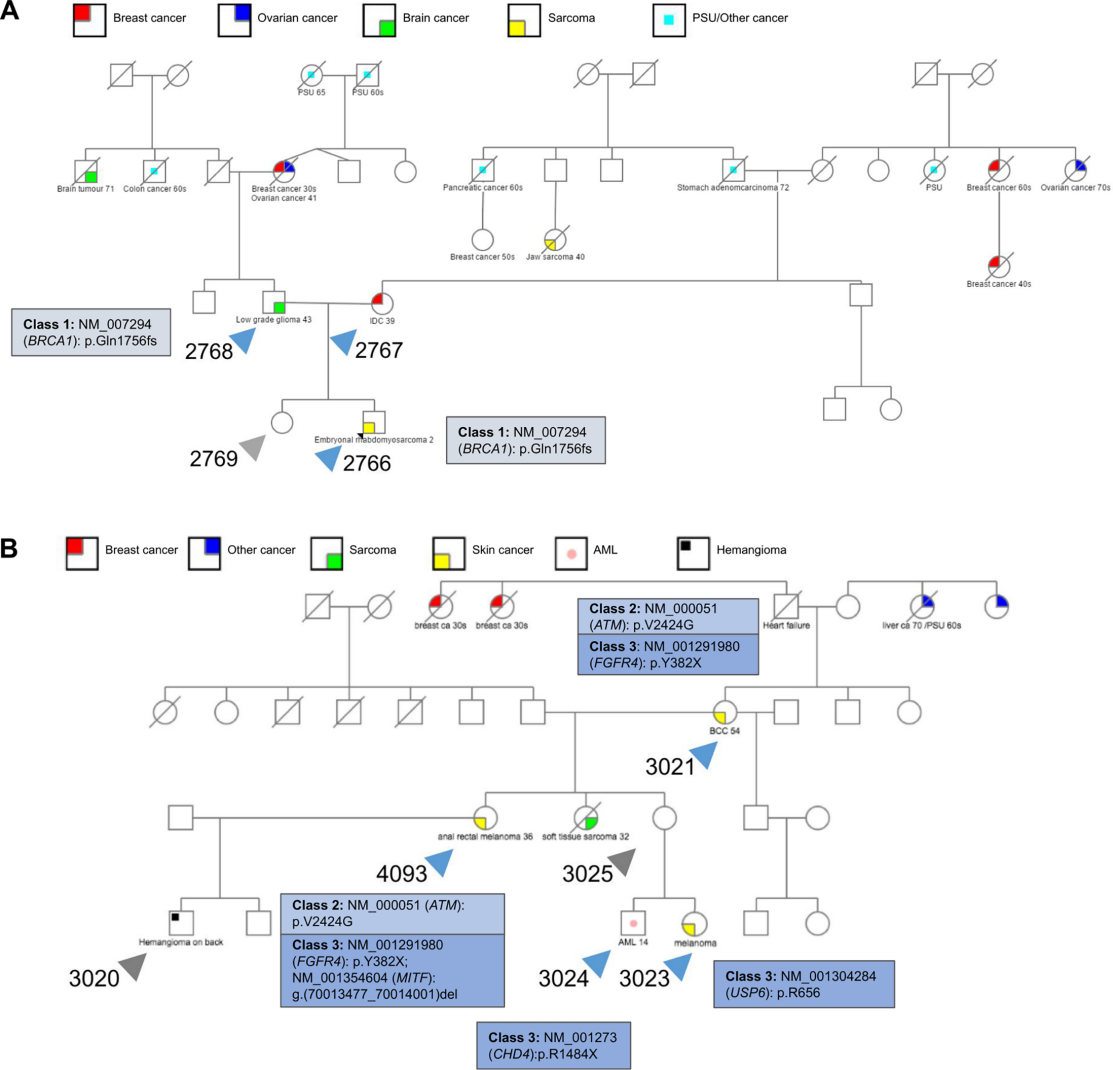
Pedigrees and class 1–3 variants for two wildtype *TP53* families that fit the classic LFS criteria. **A,** Pedigree of wildtype *TP53* family (*WT4*) that fits the classic LFS criteria with four family members sequenced, two that developed cancer and two that are cancer-free. **B,** Pedigree of wildtype *TP53* family (*WT6*) that fits the classic LFS criteria with six family members sequenced: five that developed cancer and one that is cancer-free.

Among the patients with LFS with wildtype *TP53* who developed cancer (*n* = 14)*,* we sought a genetic explanation for their cancer in the absence of a pathogenic variant *TP53* allele. A total of 14% (2/14) of the wildtype *TP53* patients harbored a P/LP variant in an autosomal dominant CPG (class 1), both of which are a *p.Gln1756fs* frameshift variant in *BRCA1* present within a single LFS family (Supplementary Table S5A). The *BRCA1* variant was initially present in the father who developed a low-grade glioma (LGG) and inherited by his son who developed an ERMS (Fig. 3A). A total of 21% (3/14) harbored a P/LP variant in an autosomal recessive CPG (class 2; Supplementary Table S5B). One such example is an *ATM p.V242G* variant that was passed down from a mother in one family (Fig. 3B) with basal cell carcinoma to her daughter with anorectal melanoma. A total of 21% (3/14) of the patients with wildtype LFS with cancer had a P/LP variant in a known cancer gene (class 3; Supplementary Table S5C and S5D). There are five such variants in the *WT6* family, a premature stop codon in *FGFR4* (*p.Y382X*), *CHD4* (*p.R148X*), and *USP6* (*p.R656X*) and a deletion in *MITF*. Interestingly, the patient who harbored a deletion in *MITF*—a gene that is well characterized in the context of familial melanoma as having an essential role in melanin synthesis in melanocytes—also developed an extremely rare and aggressive form of melanoma known as anorectal melanoma (Fig. 3B; refs. 62, 63).

Among the identified class 1–3 variants, all but one—a pathogenic variant in *XPC—*segregated with individuals that developed cancer in a given wildtype *TP53* family (Supplementary Fig. S7B – Patients 1970 and 1972). We also noticed a higher burden of P/LP variants across known cancer predisposition (class 1–2; *P* = 3.9 × 10^−4^) and cancer-associated genes (class 1–3) was associated with cancer status in wildtype *TP53* carriers (*P* = 2.3 × 10^−3^; Supplementary Fig. S4). P/LP variants in novel, candidate cancer genes (class 4) including *SF3B4* (Fig. 3B – Patient 4093)*, FMN2* (Fig. 3B – Patient 4093), and *MMP13* (Supplementary Fig. S7C – Patient 1774) are found in 21% (3/14) of patients with cancer (Supplementary Table S5E and S5F). Although the clinical significance of these variants is difficult to ascertain based on current knowledge, repeated documented cases are key in identifying novel cancer drivers and redundancies in critical signaling pathways. Finally, disease-segregating VUS in cancer genes (class 5) provide insights on an additional 7% (1/14) of the *TP*53 wildtype patients (Supplementary Table S5G), in particular a patient who developed an invasive ductal carcinoma with VUSs in *PRF1* and *BRD4* (Supplementary Fig. S7D – Patient 2767)*.*

### Leveraging Epimutations to Personalize Cancer Surveillance in *TP53* Variant Carriers

**FIGURE 4:**
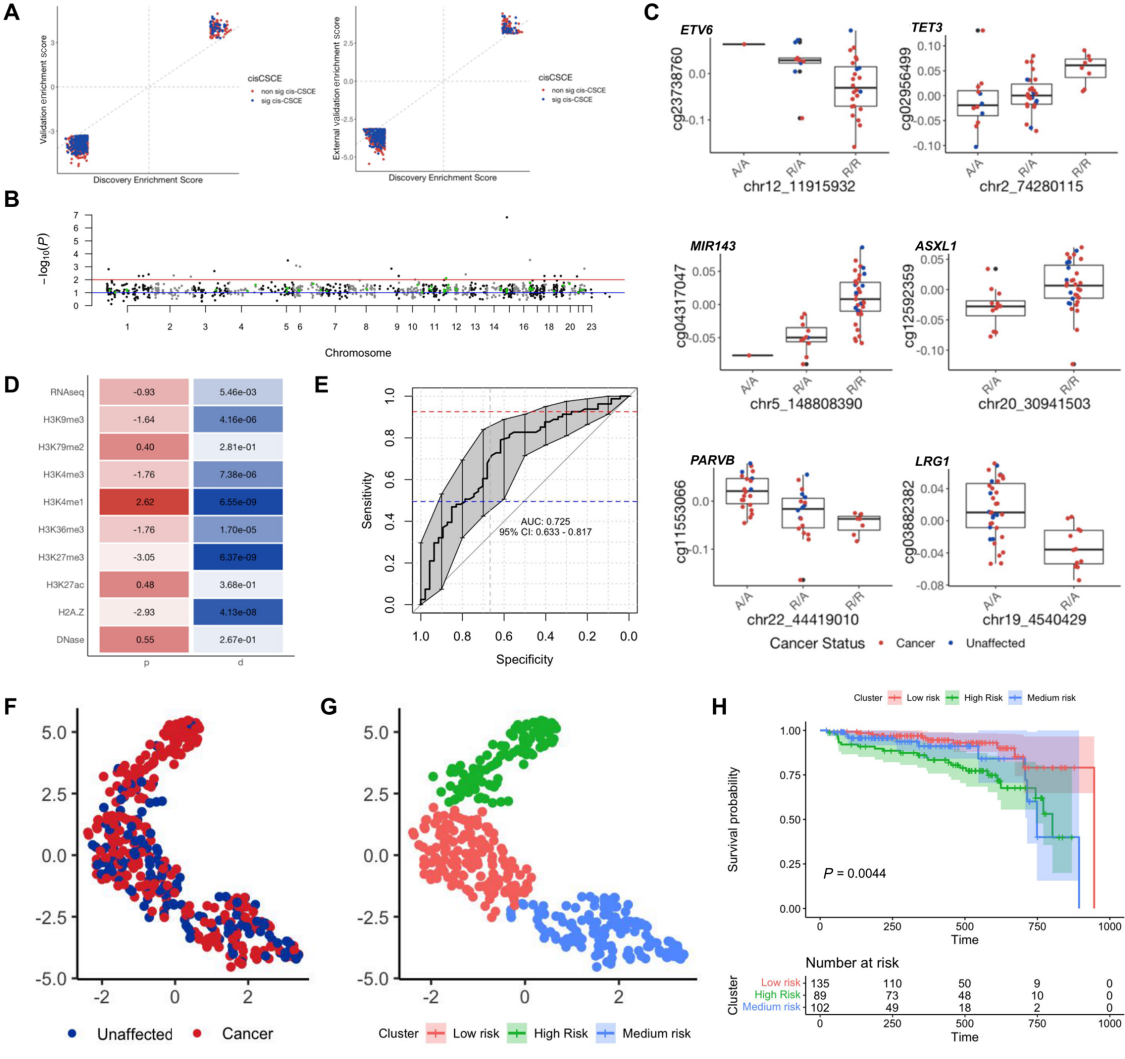
Secondary constitutional epimutations in LFS. **A,** Concordance between enrichment scores of 931 EWAS probes found significantly associated with cancer status in the discovery cohort and the validation cohort, for both the internal (left) and external (right) validation cohort. Blue = associated with cancer status and a *cis*-CSCE; Red = associated with cancer status but not a *cis*-CSCE. **B,** Manhattan plot of adjusted *P* values from meQTL association tests between the 931 EWAS probes and their most correlated rsSNP. Red line: FDR = 0.1; Blue line: FDR = 0.01; Green points: probes in cancer genes with FDR < 0.1. **C,** Top *cis*-CSCE in *ETV6, TET3, MIR143, ASXL1, PARVB*, and *LRG1.***D,** Feature enrichment heatmap of the 259 significant *cis*-CSCE loci showing adjusted *P* value (p) and effect size (d). **E,** ROC curve of the epimutation cancer risk model on the test set. **F,** UMAP projection of methylation at 259 *cis*-CSCE for all patients, colored by cancer status. **G,** UMAP projection of methylation at 259 *cis*-CSCE for all patients, colored by clusters associated with risk. **H,** Kaplan–Meier survival curve demonstrating significant differences in survival probability between the three epimutation clusters.

To identify modifiers of cancer incidence among patients with LFS with variant *TP53* in the noncoding genome, we evaluated epimutations. Using methylation data from DNA extracted from PBL of patients with LFS (*n* = 359), we identified 931 probes significantly associated with cancer incidence in our discovery cohort and validated them in an internal and external cohort (Fig. 4A); 4.2% (39/931) of the probes lie in cancer genes (Supplementary Table S6). A total of 27.8% (259/931) of the probes were *cis* methylation quantitative trait loci (meQTL; FDR < 0.05; Fig. 4B). These 259 loci represent *cis* cancer-associated secondary constitutional epimutations (*cis*-CSCE)—inherited SNPs in *cis* with methylation marks associated with cancer (52, 53).

Similar to the pathway analysis performed on the genetic variants, pathway analysis of the genes that the 259 significant *cis*-CSCE reside in identified the p53 pathway as being the most significantly enriched pathway (FDR = 0.04). Eight of the *cis*-CSCE lie in cancer genes on our Tier 3 list, including *ASXL1* (*q_CSCE_ = 1.04 × 10^−^^2^;rho_CSCE_ =* −*0.371*) and *ETV6* (*q_CSCE_ = 2.41 × 10^−^^2^;rho_CSCE_ = 0.386;*Fig. 4C). A total of 50.6% (131/259) were associated with increased methylation with more copies of the alternate allele. 86.5% (224/259) of *cis*-CSCE were associated with an increased cancer risk upon hypomethylation. The remaining 13.5% (35/259) of *cis*-CSCE were positively associated with cancer risk. The majority of the *cis*-CSCE lie within the open sea in intergenic regions or within the body of a gene (Supplementary Fig. S8). Feature enrichment analysis at the genomic loci of the significant *cis*-CSCE showed enrichment of H3K4me1 marks, which are associated with the presence of enhancers (Cohen *d* = 2.62, *P* = 6.55 × 10^−9^; Fig. 4D).

We then leveraged the 259 *cis*-CSCE to train a random forest model to predict cancer risk in patients with LFS. Our model achieved an AUROC of 0.725 (0.633–0.810) and area under the precision recall curve (AUPRC) of 0.783 (0.775–0.792) on the test set (Fig. 4E). The optimal specificity of our model was determined as 42.0% (Materials and Methods), and corresponds to a sensitivity and positive predictive value of 85.1% and 46.0%, respectively, on the test set. Interestingly, our model was able to correctly classify an additional 22.4% (11/49) of the germline *TP53* carriers that developed cancer for whom we were unable to identify a class 1–3 variant. However, it also misclassified 13 patients with a class 1–3 variant as cancer-free, suggesting that in the presence of a strong driver, epimutations may have a dampened role in contributing to elevated cancer risk. K-means clustering of the UMAP projection of the 259 *cis*-CSCE revealed three distinct clusters, whereby cluster membership was significantly associated with cancer status (Fig. 4F; *χ*^2^ test, *P* = 4.4 × 10^−7^) and survival differences (Fig. 4G and H; *P* = 0.0044). There was no clear relationship between the clusters and age of cancer onset or tissue type (Supplementary Fig. S9).

### *LEF1* and the WNT Signaling Pathway as Modifiers Associated with Decreased Cancer Risk in *TP53* Variant Carriers

The pathway enriched for genetic variants in the cancer-free LFS family members was the WNT β-catenin signaling pathway (FDR = 0.04; Supplementary Fig. S3). It has been previously recognized that the loss of function of p53 results in the upregulation of downstream WNT target genes (*TCF/LEF* target genes, *CD44, MMP7, MYCN*), suggesting that p53 represses canonical WNT signaling (64). Among the patients with LFS with variant *TP53*, the WNT β-catenin signaling pathway was significantly mutated in the germline of 23% (3/13) of individuals who were cancer-free compared with 2% (1/49) of patients that developed cancer (Wilcoxon rank-sum test, FDR = 0.08). Likewise, individuals with a modifier in the WNT signaling pathway had better overall survival than those without a WNT signaling modifier (Fig. 2C). In one family (Supplementary Fig. S5B), the two unaffected family members (Patients 2815 and 2565) harbored a pathogenic variant in *LEF1* that was absent in their cancer-affected relatives, suggesting its potential protective role*. LEF1* is a transcription factor that activates oncogenes such as cyclin D1 and MYC to mediate cellular transformation and it has been shown that in the presence of a pathogenic variant in *TP53,* there are elevated levels of *LEF1* that drive aberrant WNT signaling (64–66).

**FIGURE 5:**
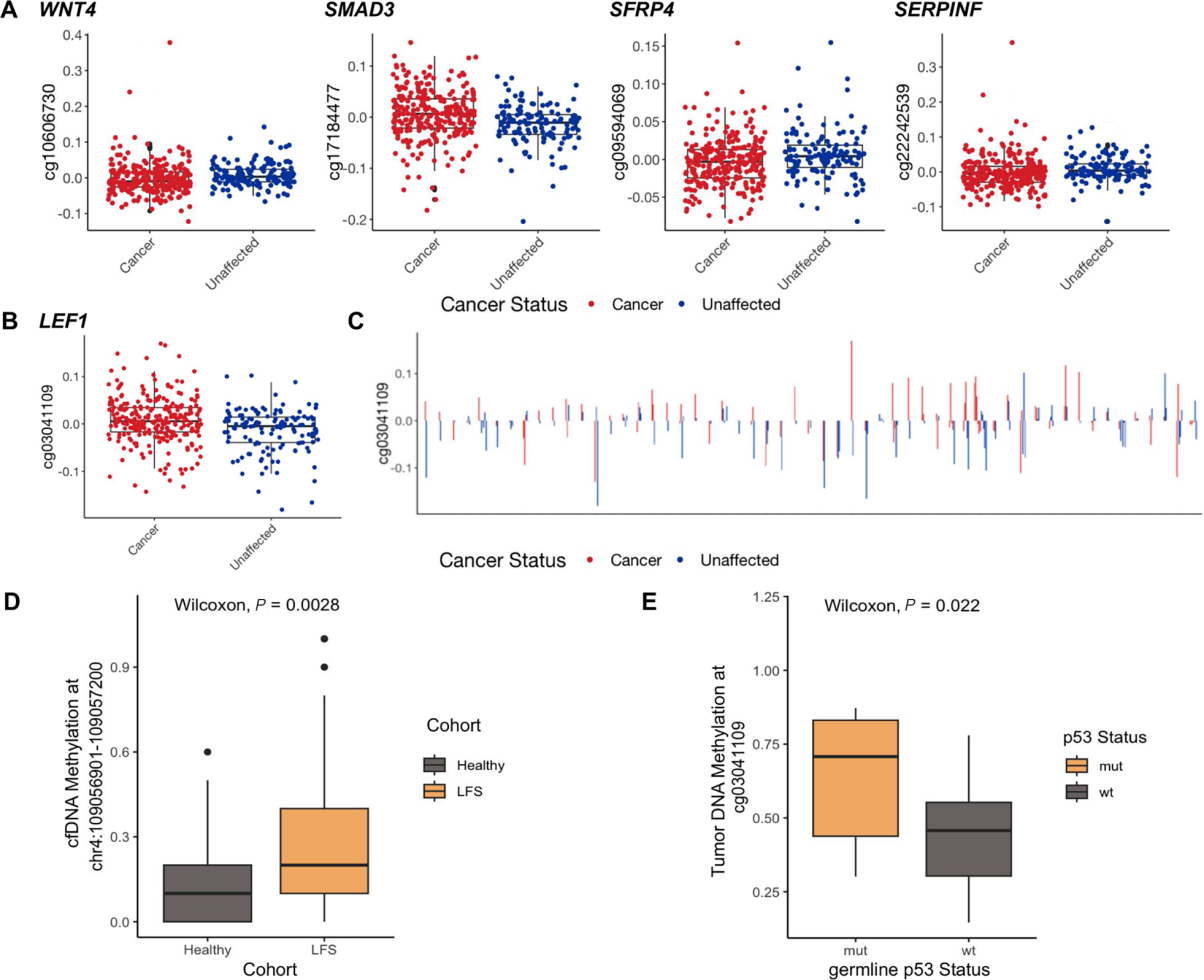
Differential regulation of WNT signaling and *LEF1* in LFS. **A,** Probes in the WNT signaling pathway (*WNT4, SMAD3, SFRP4, SERPINF*) significantly associated with cancer status. **B,** Probe in the body of *LEF1* (cg03041109) associated with cancer status (FDR = 0.07). **C,** Methylation of probe in body of *LEF1* (cg03041109) across members within each LFS family. Probands almost unanimously have higher *LEF1* methylation than their unaffected family members. **D,** Plasma cfDNA methylation of the body o*f LEF1* (chr4:109056901-109057200), between patients with LFS and HBCs. **E,** Tumor methylation of the body of *LEF1* (cg03041109) in CPTs, with and without a germline variant in *TP53*.

Among the 931 methylation probes found significantly associated with cancer status, several were located within genes involved in the WNT signaling pathway (Fig. 5A). This included the probe *cg03041109* in the body of *LEF1,* which was significantly associated with cancer status (FDR = 0.02; Fig. 5B). We found probands almost universally exhibited higher *LEF1* methylation than their unaffected family members (Fig. 5C). This further suggests the presence of molecular differences in the WNT signaling pathway between patients with LFS with and without cancer.

To further explore this, we evaluated methylation at the genomic location of *cg03041109* in tumors and plasma cell-free DNA (cfDNA) of individuals with and without a germline variant in *TP53*. We found cfDNA of individuals with LFS were hypermethylated at *LEF1* compared with HBCs with wildtype *TP53* (Wilcoxon rank-sum test, *P* = 2.8 × 10^−3^*;*Fig. 5D). We also compared *LEF1* methylation across CPTs—a characteristic LFS tumor—with or without a germline *TP53* variant. Similar to PBLs and cfDNA of cancer-affected patients with LFS, we found increased *LEF1* methylation in CPT with a germline *TP53* variant compared with those with wildtype *TP53* (Wilcoxon rank-sum test, *P* = 0.022*;*Fig. 5E).

To assess whether *LEF1* methylation was associated with prognosis across paediatric cancers, we compared methylation of *LEF1* in blood (control), normal kidney, NRs, WT with favorable histology, and WT with anaplastic histology across two cohorts, one from Toronto, Canada (57) and the other from the United Kingdom (58). Notably, anaplasia in WT is characteristic of tumors with a somatic *TP53* variant and is associated with poor prognosis (67). We found WT with anaplasia were significantly hypomethylated at *LEF1* compared with blood (p_Toronto_ = 6.4 × 10^−7^), normal kidney (p_Toronto_ = 7.2 × 10^−8^, p_UK_ = 5.3 × 10^−6^), NR (p_UK_ = 2.4 × 10^−3^) and WT with favorable outcomes (p_Toronto_ = 4.5 × 10^−2^, p_UK_ = 5.1 × 10^−3^; Supplementary Fig. S10A). To evaluate whether this pattern was reflected in adult cancers lacking a germline *TP53* variant, we compared *LEF1* methylation in tumors with or without a somatic *TP53* variant in LFS-associated cancers from The Cancer Genome Atlas. We found variable patterns of *LEF1* methylation across tumors, with or without a somatic *TP53* variant (Supplementary Fig. S10B). For instance, tumors with a somatic *TP53* variant had increased methylation in LGG, but decreased methylation in sarcomas and breast cancers, compared with tumors with wildtype *TP53*.

## Discussion

Hereditary cancers comprise 10% of all cancer incidence worldwide (68), of which it is estimated that up to 16% of childhood cancers are associated with pathogenic germline variants in CPG (43, 69, 70); in actuality these figures are likely much greater. Although in recent years, the discovery of common variants with small effect sizes has resulted in a paradigm shift of our understanding of cancer as a monogenic disease to a multifactorial disease, hereditary cancer syndromes, including LFS, have historically been considered monogenic diseases in which a single gene has been causally implicated. As a result, other contributing factors are frequently overlooked, even with variable cancer penetrance and phenotypes.

In this study, we present combined germline WGS and methylation data on a large multi-institutional cohort of individuals who fit the clinical definitions of LFS. We have demonstrated the novel utility of genetic and epigenetic data for personalized stratification of patients with LFS by two important clinical outcomes: cancer risk and overall survival. In individuals that lack a germline *TP53* variant we identified alternative cancer-causing aberrations in 57.1% (8/14) of individuals with wildtype *TP53* who developed cancer; this will enable the diagnosis of LFS in patients that would have been otherwise missed through standard screening procedures. Among variant *TP53* carriers, we found 38.8% (19/49) who developed cancer harbored an additional P/LP variant in another cancer gene. Of note, modifier variants in the WNT signaling pathway were associated with decreased cancer incidence and improved survival outcomes in variant *TP53* carriers*.* Furthermore, we evaluated the noncoding genome and methylome and identified inherited epimutations in genes including *ASXL1*, *ETV6,* and *LEF1* that confer increased cancer risk. Leveraging these epimutations, we built a machine learning model that can predict cancer risk in patients with LFS with an AUROC of 0.725 (0.633–0.810). Taken together, this will enable the personalized stratification of patients with LFS based on the (i) the presence of a P/LP variant in a known cancer gene, (ii) predicted probability of developing cancer using our epimutation cancer risk model, (iii) epimutation cluster analysis, (iv) presence of a P/LP variant in the WNT signaling pathway, and (v) *LEF1* methylation (Table 2). It clarifies the genomic basis for the phenotypic variability in LFS and highlights the immense benefits of expanding genetic and epigenetic testing of patients with LFS beyond *TP53*.

**TABLE 2:** Genomics informed risk stratification guidelines for patients with LFS with a germline *TP53* variant

High-risk variant *TP53* carrier	Low-risk variant *TP53* carrier
P/LP variant in a cancer gene	P/LP variants in the WNT signaling pathway
High probability of developing cancer using our epimutation cancer risk model	Low probability of developing cancer using our epimutation cancer risk model
High-risk epimutation cluster	Low-risk epimutation cluster
*LEF1* hypermethylation	*LEF1* hypomethylation

Approximately 30% of individuals who fit the clinical criteria used to diagnose LFS lack a pathogenic germline variant in *TP53* (71). This suggests the involvement of other genetic and/or epigenetic mechanisms that contribute to cancer development in LFS. Candidate gene approaches have been taken to consider other relevant genetic causes in individual cases of LFS, proposing the role of alternative genes including *CHEK2* (13–15)*, TP63* (15)*, BRCA1* (16, 17)*, BRCA2* (18), and *CDKN1A* (12). One study performed whole-exome sequencing of two index cases with cardiac angiosarcoma from a *TP53*-negative LFS family and identified an inherited pathogenic variant in *POT1* (19). However, these studies fail to provide a comprehensive view of the germline genome in patients with LFS lacking a pathogenic variant in *TP53*. In addition, the majority of studies to-date have evaluated a single gene or utilized a limited panel of clinically recognized CPG (i.e., class 1) to screen for inherited cancer susceptibilities. In this study, we performed a genome-wide analysis of patients with LFS lacking a pathogenic germline variant in *TP53* and adopted a tiered approach to evaluate cancer risk. In doing so, we identified alternative cancer-associated genetic aberrations (class 1–3) in 57% (8/14) of cancer-affected individuals diagnosed with LFS but lacking a pathogenic germline variant in *TP53*. This suggests that it is crucial that genetic testing in patients who fit the clinical definition of LFS be expanded beyond *TP53* to permit the prospective management of these patients and their families. In addition to known cancer-associated variants, we identified P/LP variants in the novel candidate cancer genes *SF3B4*, *FMN2,* and *MMP13* (class 4)*.* Although promising, the P/LP variants we identified in novel candidate cancer genes (class 4) and disease-segregating VUSs in cancer-associated genes (class 5) require experimental validation to functionally characterize these alterations and determine their oncogenic capability.

In the 1980s, Vogelstein and Kinzler proposed a model for colorectal tumorigenesis that required four sequential alterations involving the activation of an oncogene coupled with the loss of three tumor suppressors (61). More recently, in a pan-cancer analysis, cancer genomes were found to contain driver variants affecting an average of four to five genes (70). Previous studies of pediatric cancer cases have also demonstrated that pathogenic germline variants in different genes can synergistically drive tumorigenesis (72). In line with these findings, we observed that patients with LFS harboring a P/LP *TP53* variant with a co-occurring germline hit in another cancer gene (class 1–3) were associated with increased cancer incidence and decreased cancer survival compared with variant *TP53* carriers lacking any class 1–3 variants. We also found that *BRCA1* was the only autosomal dominant CPG (i.e., class 1), besides *TP53,* that harbored a P/LP variant. However, several other relevant cancer genes harbored P/LP variants that would have been otherwise overlooked, some of which are therapeutically actionable.

Furthermore, it is estimated that approximately 20% of patients with LFS with a pathogenic germline variant in *TP53* never develop cancer. Understanding the reason for this apparent resiliency and the incomplete penetrance in LFS is crucial for risk stratification and ultimately informing patient management. We found variants in the WNT signaling pathway were associated with reduced cancer incidence and improved cancer survival compared with variant *TP53* carriers lacking variants in the WNT signaling pathway, and even more so compared with those with at least one class 1–3 variant. Moreover, *LEF1* hypermethylation was associated with increased cancer risk in patients with LFS with a germline *TP53* variant. However, in WT and adult tumors with a somatic *TP53* variant, hypomethylation of LEF1 was associated with poor prognosis, suggesting the relationship between *LEF1* and patient outcomes is dependent on the timing of the *TP53* variant (i.e., early germline event vs. later somatic event). Previously published work supports a model whereby aberrant WNT signaling abrogates p53 dysfunction (64), and future work should investigate this mechanism in the context of germline events.

Epigenetics has often been proposed as a mechanism to explain the “missing” causality and heritability of cancer (73–75). DNA methylation of PBL—as a proxy for the germline—is implicated in the transcriptional modulation of several cancers at regulatory regions of tumor suppressors and oncogenes (76–79). Susceptibility loci are enriched in these regulatory regions and can modulate the epigenome (80–82). Inherited methylation marks linked to genetic variants are referred to as secondary constitutional epimutations—formed by an initial genetic variant, which can then be inherited by offspring through genetic transmission of the initial variant or epigenetic transmission of the subsequent epimutation (83, 84). Previous research suggests patients with LFS with germline *TP53* variants possess distinct PBL methylation signatures in comparison to their sporadic counterparts, suggesting a role for epimutations in LFS tumorigenesis (9). In our study, we leveraged the noncoding genome and methylation of patients with LFS and identified 931 methylation probes that are associated with increased cancer risk, of which 259 were associated with a *cis*-SNP. These epimutations suggest another mechanism by which the phenotype of patients with LFS is altered to favor a malignant state. However, it is difficult to discern the complementarity of the P/LP variants and epimutations, in an unbiased manner, without an additional validation cohort for which both WGS and methylation data are available.

We note, in our identification of *cis*-CSCE, the majority of PBL samples used for methylation profiling were collected from individuals that developed cancer, at or following diagnosis. While little is understood about the effects of chemotherapeutic drugs on DNA methylation of PBL, it remains an important consideration. To address this, we first removed broad, cancer- and treatment-associated signals from the methylation data. In previous work, Lemire and colleagues identified the absence of a global or local effect of chemotherapy on methylation in individuals with colorectal cancer (85). In contrast, Yao and colleagues found 4.2% of the CpG sites measured using the Illumina 450K methylation array underwent significant changes in patients with breast cancer after chemotherapy (86). Only 1.2% (7/568) of the probes associated with chemotherapy in Yao and colleagues overlapped with the 931 EWAS probes significantly associated with cancer status in variant *TP53* carriers. Furthermore, we found no methylation probes significantly associated with systemic treatment status among variant *TP53* carriers. Although this suggests chemotherapy is not a confounder in our analysis, reverse causation remains a potential limitation and further prospective studies are required.

Our study captures a snapshot of the clinical heterogeneity in LFS. To gain a comprehensive understanding of the germline landscape of LFS, further collaborative, prospective genetic and epigenetic profiling of similarly large cohorts of patients is crucial. Overall, our findings highlight the potential of expanding genetic and epigenetic testing of LFS patients beyond *TP53*, as *TP53* alone does not explain the vast clinical heterogeneity in LFS. It also further necessitates the dissociation of hereditary cancer syndromes as single gene disorders given the existence of other factors that contribute to a cancer phenotype. Furthermore, these diseases need to be studied in a holistic manner as opposed to through the lens of a single gene. Future surveillance and treatment of hereditary cancer syndromes, like LFS, should incorporate genome-wide germline molecular profiling in order to provide personalized patient management.

## Supplementary Material

Supplementary Figures S1-S12 Supplementary Figures

Supplementary Data S1 LFS Cohort

Supplementary Data S2 Tier 1-3 Gene Lists

Supplementary Data S3 1000G Variants

Supplementary Data S4 KiCS Variants

Supplementary Data S5 LFS Variants

Supplementary Data S6 cisCSCE
