# Supplementary material for: Multiple Germline Events Contribute to Cancer Development in Patients with Li-Fraumeni Syndrome
Source: Cancer Res Commun. 2023 May 1;3(5):738–54. doi: 10.1158/2767-9764.CRC-22-0402 (PMC10150777; doi:10.1158/2767-9764.CRC-22-0402)

Supplementary Figures

Supplementary Figure S1. Association of age of cancer onset (months) with the presence of a class 1-3 variant, stratified by p53 status. Each point represents a LFS patient that developed cancer with WGS.

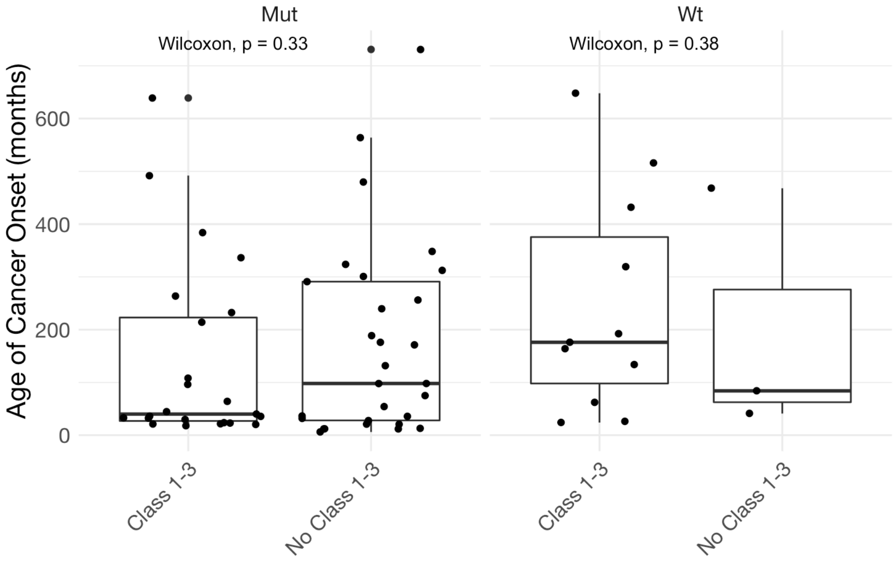

Supplementary Figure S2. The number of patients with therapeutically actionable variants by the class of drug: EZH2 inhibitors (EZH2i), growth tyrosine kinase inhibitors (TKI), PARP inhibitors (PARPi), MTOR inhibitors (MTORi).

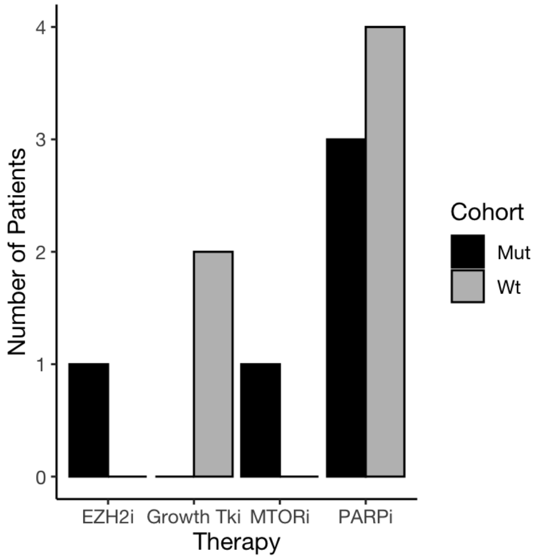

Supplementary Figure S3. Pathway analysis by cancer status and p53 status. (A) Pathogenic variants by hallmark pathways, stratified by cancer status: cancer (left), unaffected (right). For each pathway, the number of alterations is displayed in the barplot (right), along with the percentage of the cohort that harbours an alteration in that pathway (left). Pathway_qval (left) indicates the q-value for an enrichment test performed in that cohort. (**B)** Pathogenic variants by hallmark pathways, stratified by p53 status: variant (left), wildtype (right). For each pathway the number of alterations is displayed in the barplot (right), along with the percentage of the cohort that harbours an alteration in that pathway (left). Pathway_qval (left) indicates the q-value for an enrichment test performed in that cohort.

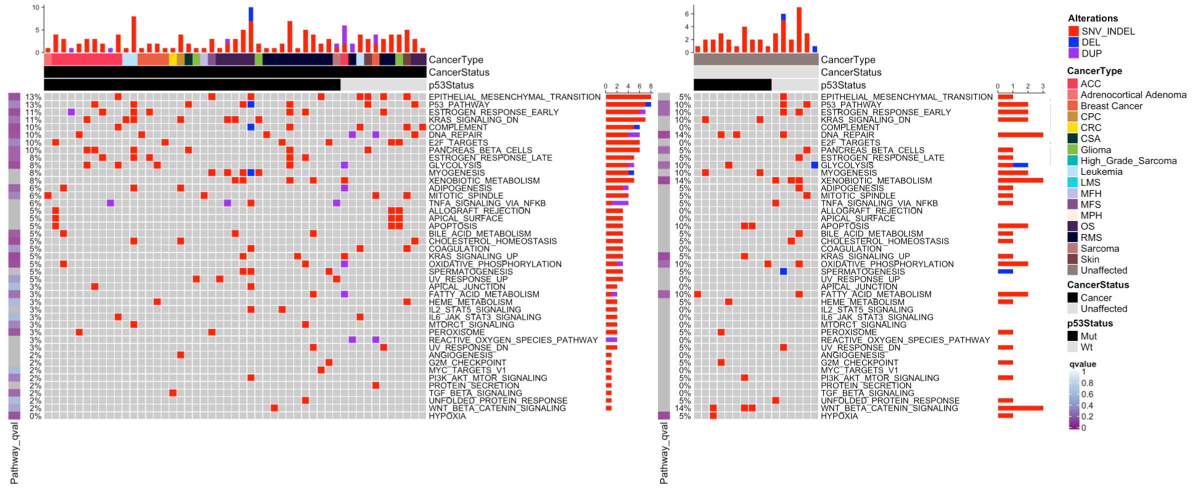

Supplementary Figure S4. The number of variants for pathogenicity Class 1-5 in (**A)** individuals that developed cancer compared to unaffected individuals, stratified by *TP53* status. **(B)** individuals with variant *TP53* compared to wildtype *TP53*, stratified by cancer status. Based on the premise that variants in the WNT signaling pathway are associated with decreased cancer incidence in LFS, these variants were removed from the comparisons.

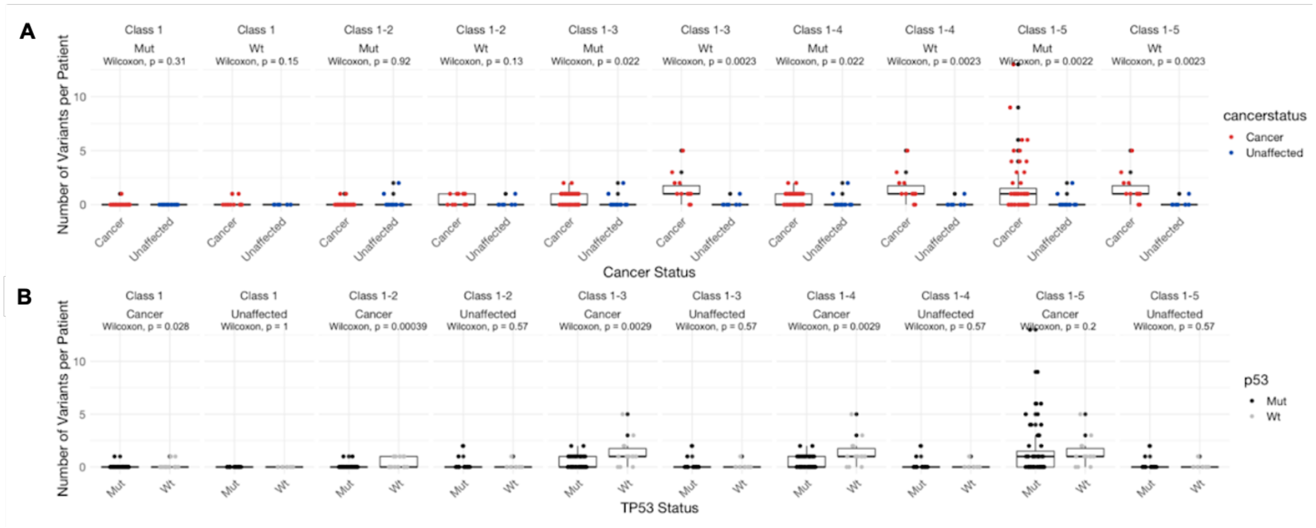

Supplementary Figure S5. Mutant (MUT) *TP53* LFS Pedigrees. Pedigrees are annotated with individuals that were sequenced indicated using an arrow (cancer-free=grey; cancer=blue). The shape indicates sex (male=square; female=circle) and colour indicates cancer type.

A. MUT7 (LFS - Chompret)

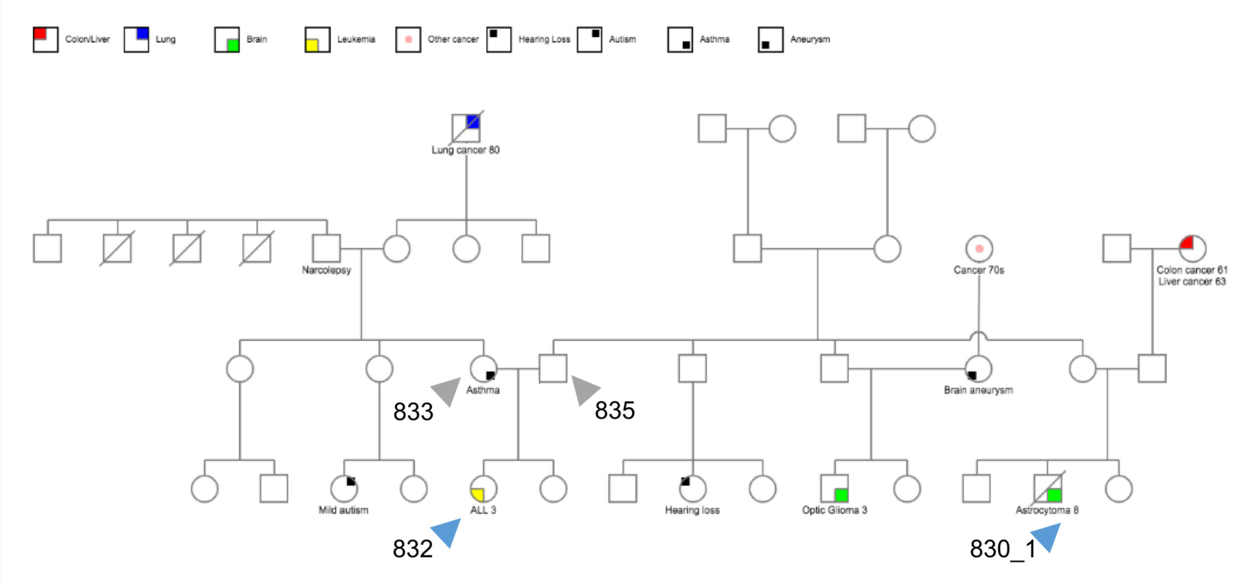

B. MUT2 (LFS - Chompret)

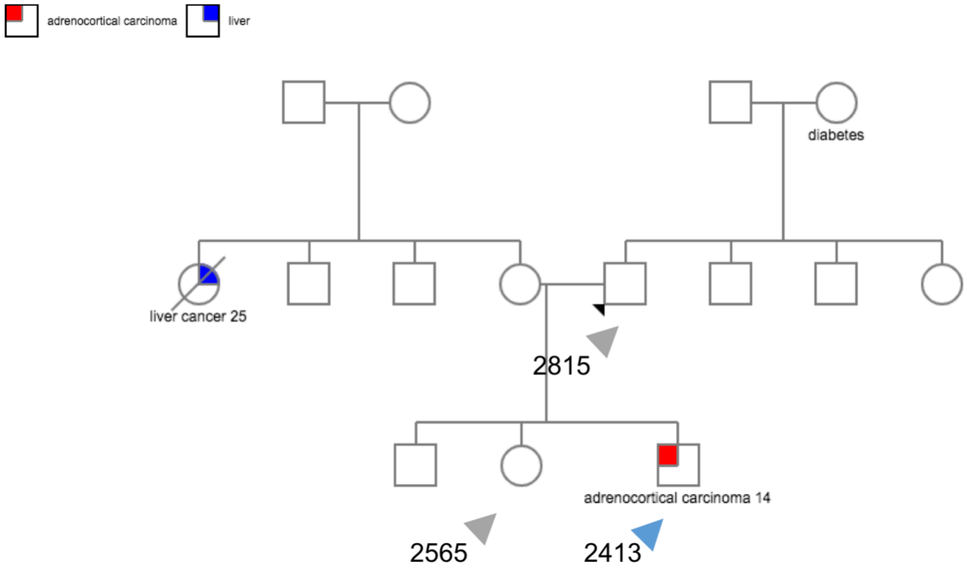

C. MUT4 (LFS - Chompret)

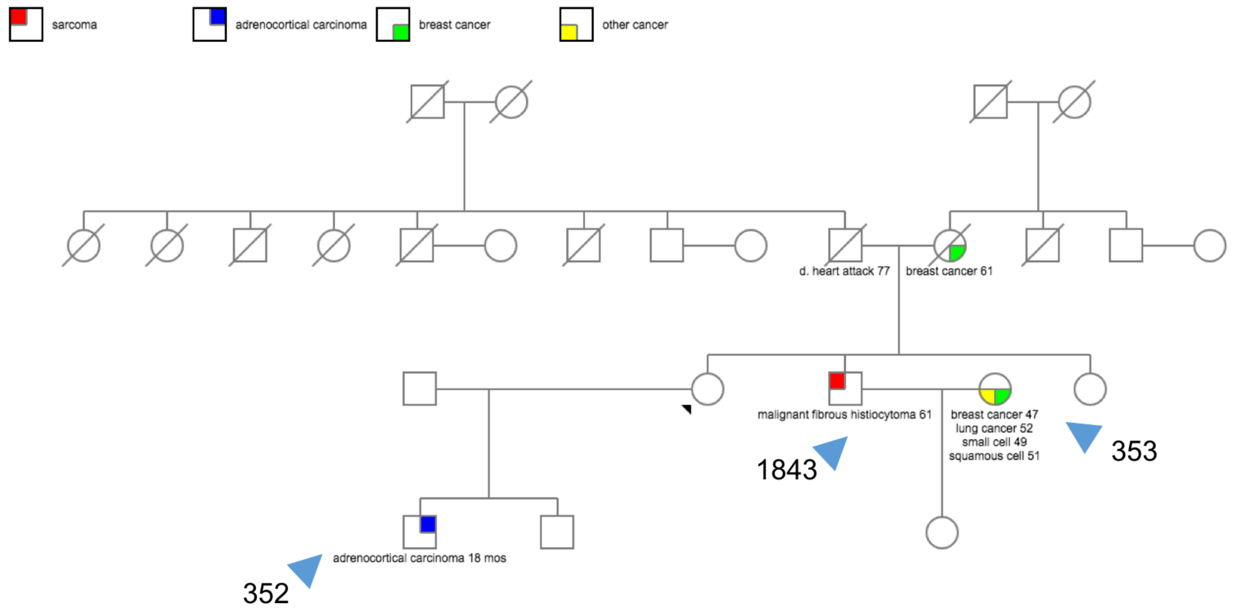

D. MUT9 (LFS - Chompret)

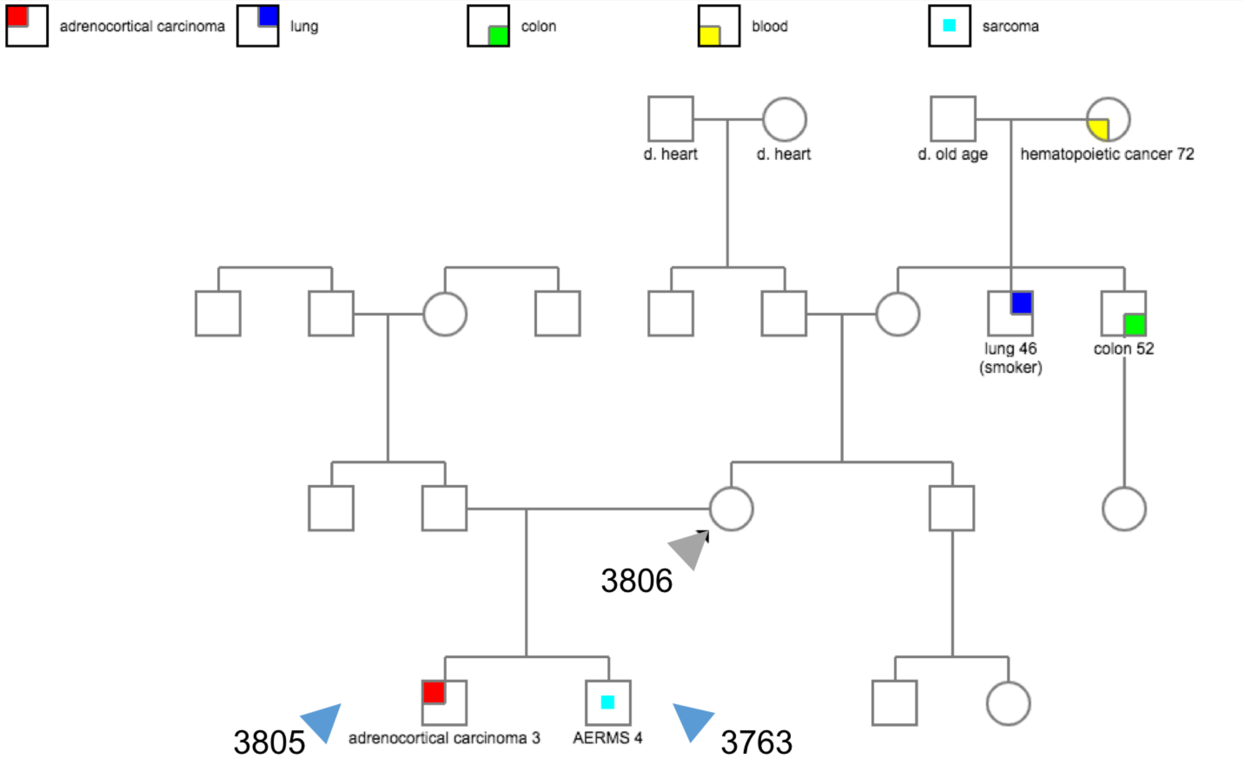

E. MUT35 (LFI)

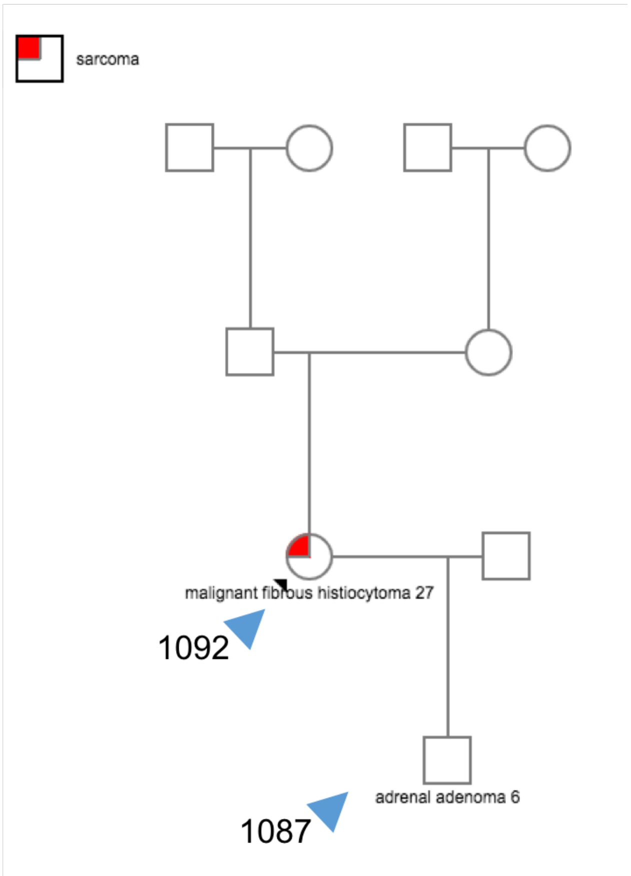

F. MUT44 (LFS - Chompret)

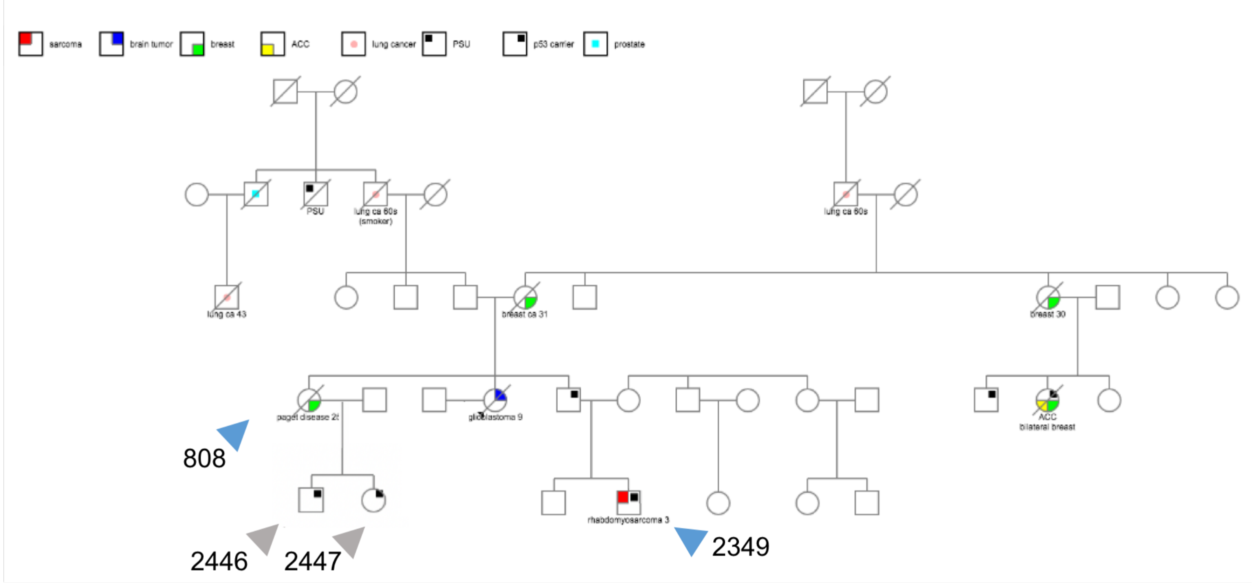

G. MUT48 (LFS - Chompret)

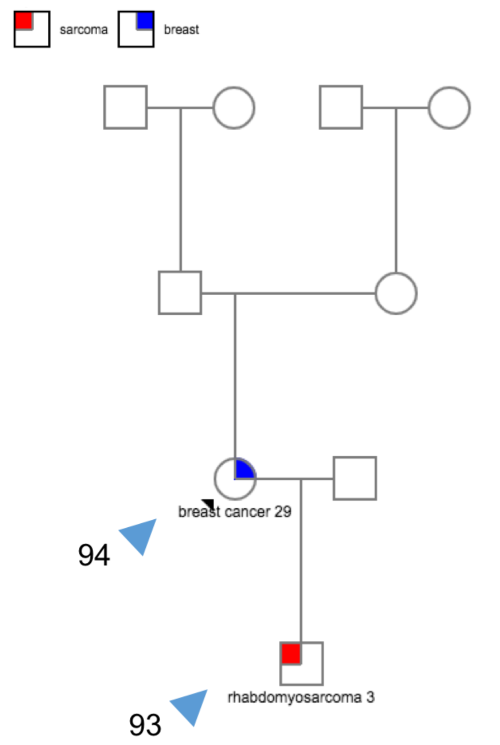

H. MUT52 (LFS - Chompret)

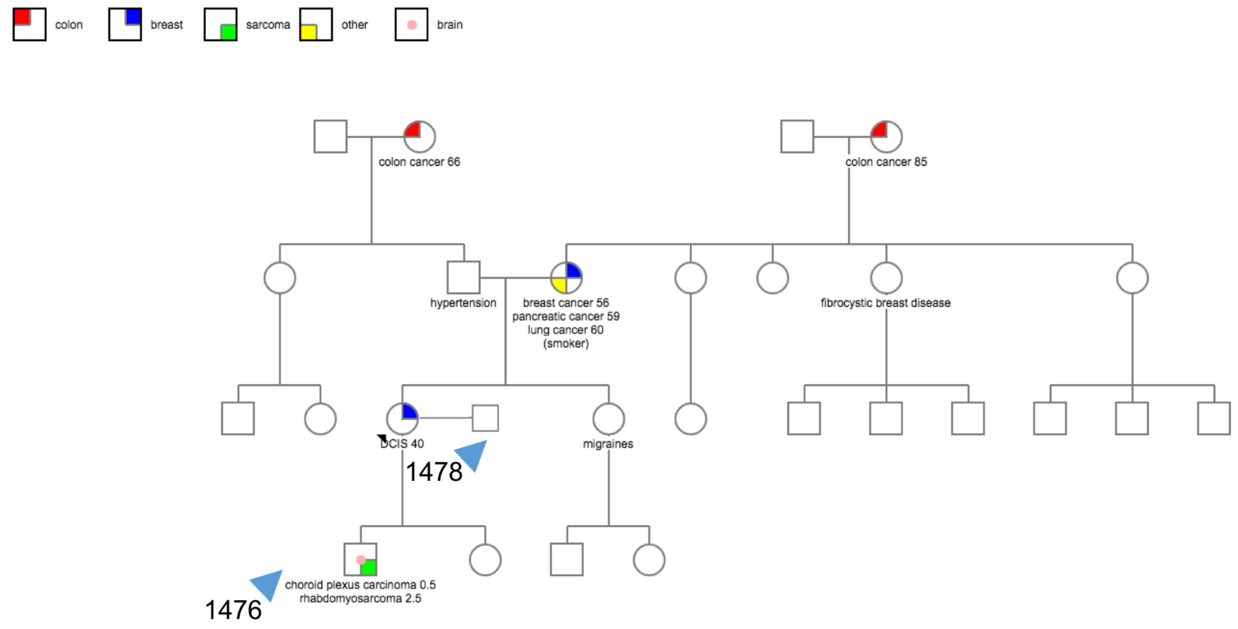

I. MUT67 (LFS - Chompret)

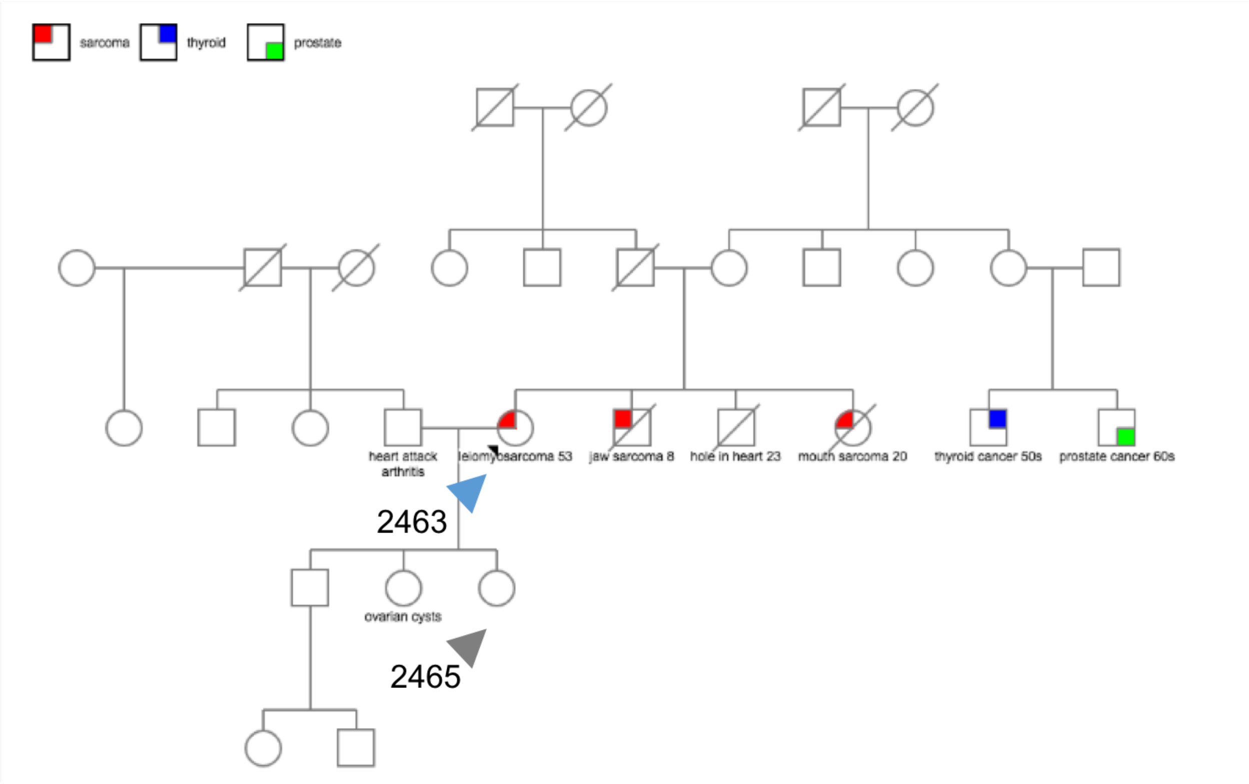

J. MUT76 (LFS - Classical)

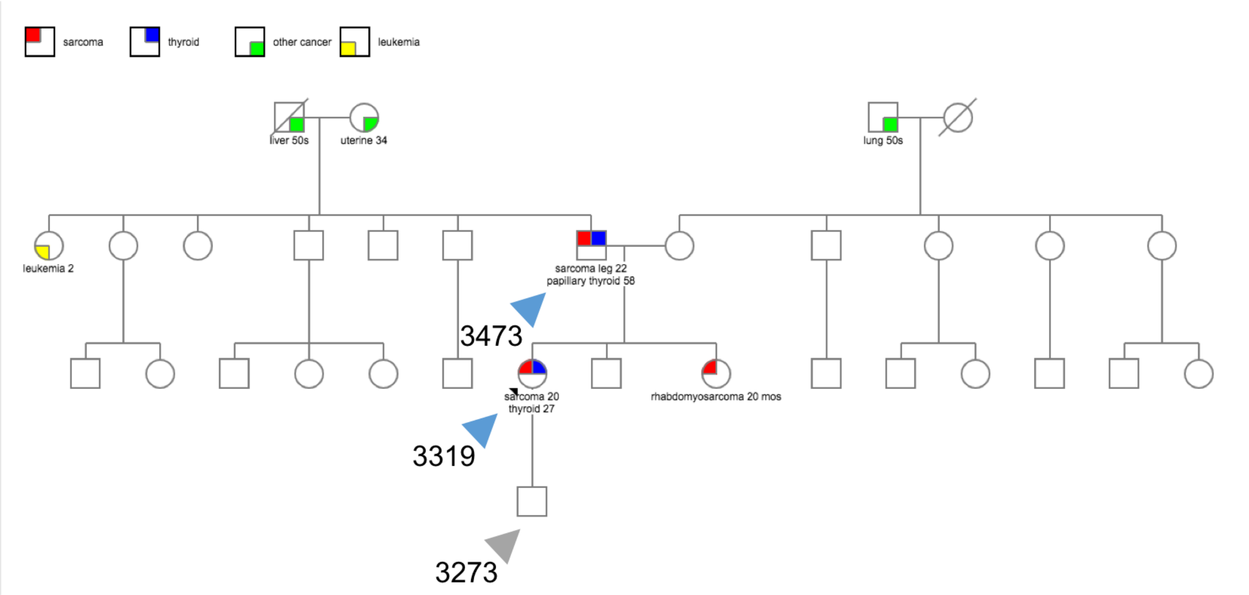

K. MUT79 (LFS - Classical)

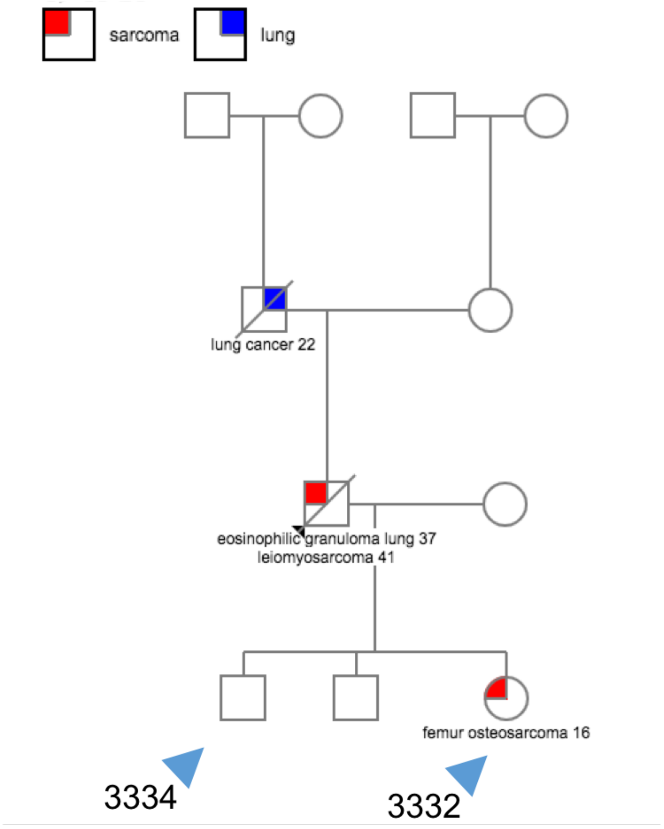

L. MUT83 (LFS - Chompret)

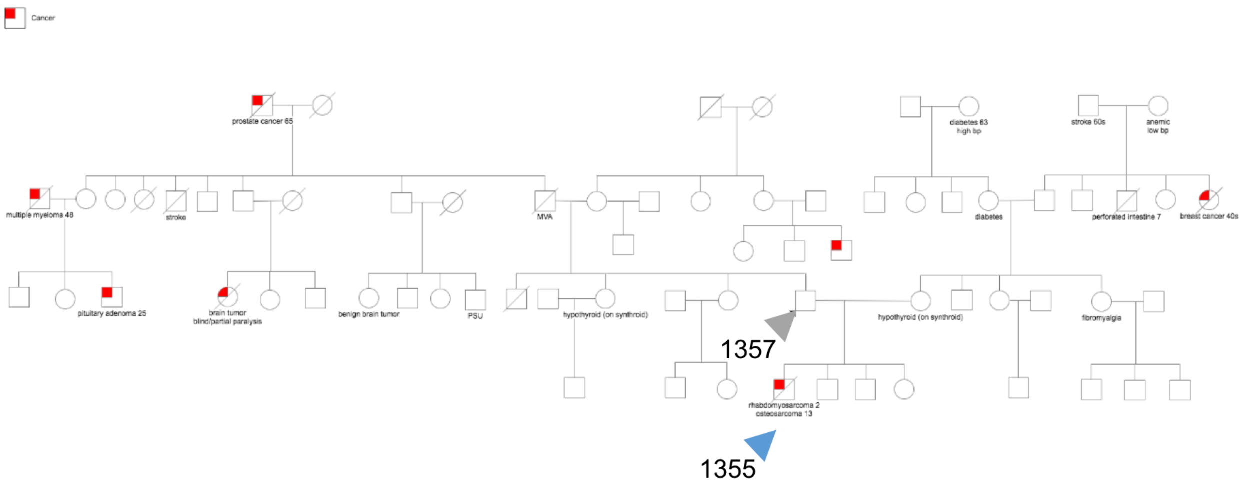

Supplementary Figure S6. The number of P/LP variants (y-axis) in wildtype versus variant LFS (x-axis), for pathogenicity Class 1-5 (Methods), stratified by cancer status. Based on the premise that variants in the WNT signaling pathway are associated with decreased cancer incidence in LFS, these variants were removed from the comparisons.

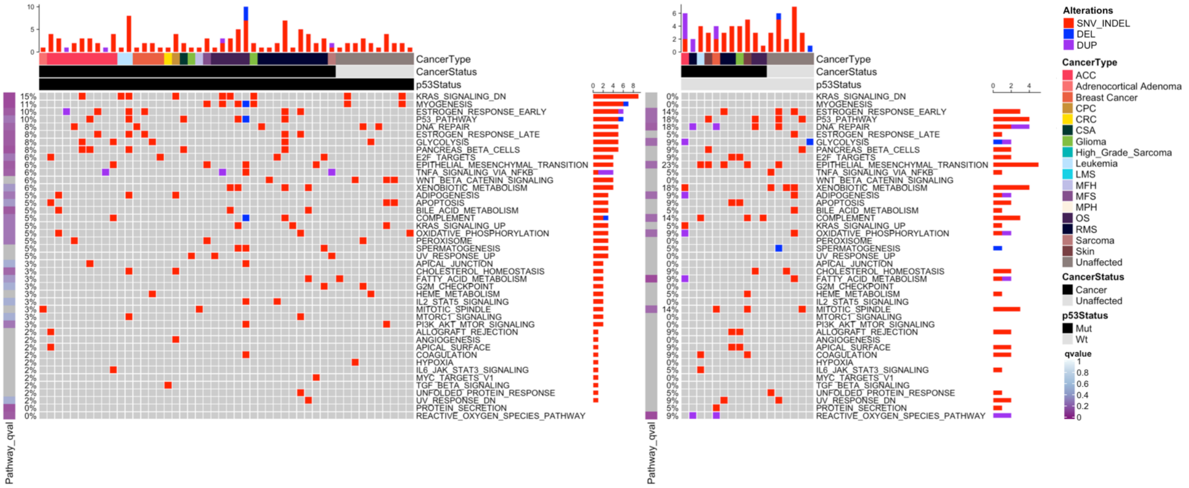

Supplementary Figure S7. Wildtype (WT) TP53 LFS Pedigrees. Pedigrees are annotated with individuals that were sequenced indicated using an arrow (cancer-free=grey; cancer=blue), whereby colour indicates cancer type and shape indicates sex (male=square; female=circle).

A. WT1 (LFS - Chompret)

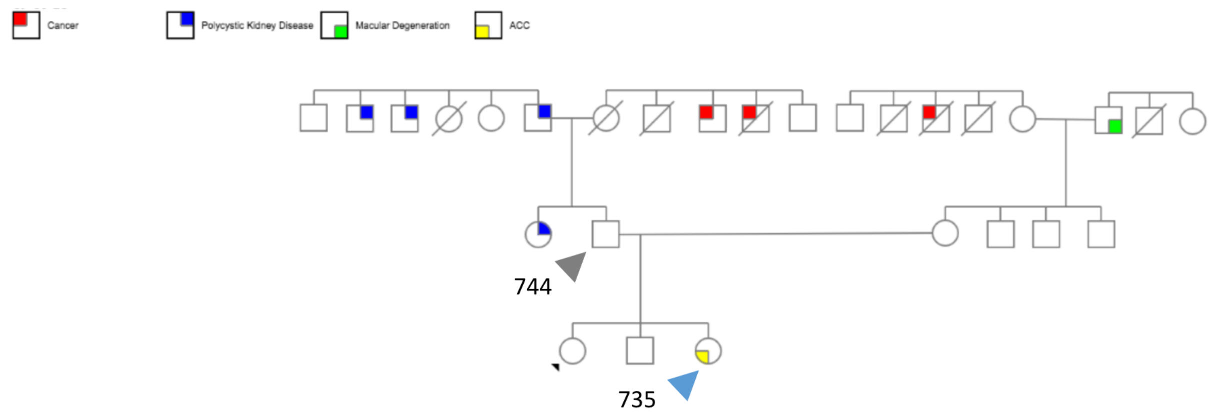

B. WT2 (LFS - Chompret)

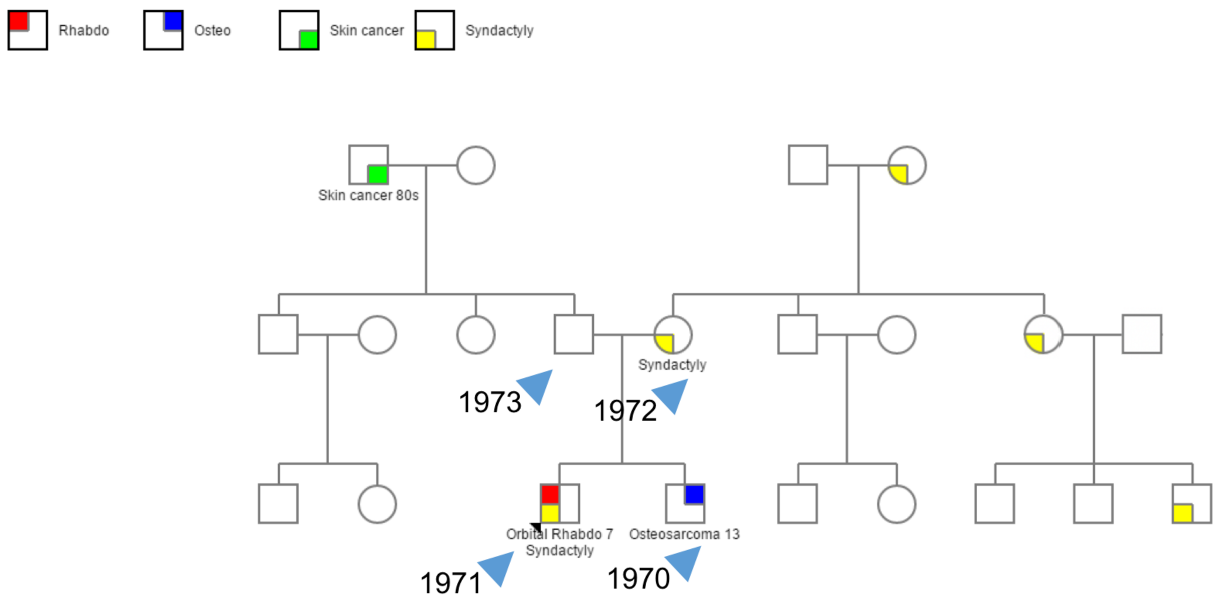

C. WT3 (LFS - Chompret)

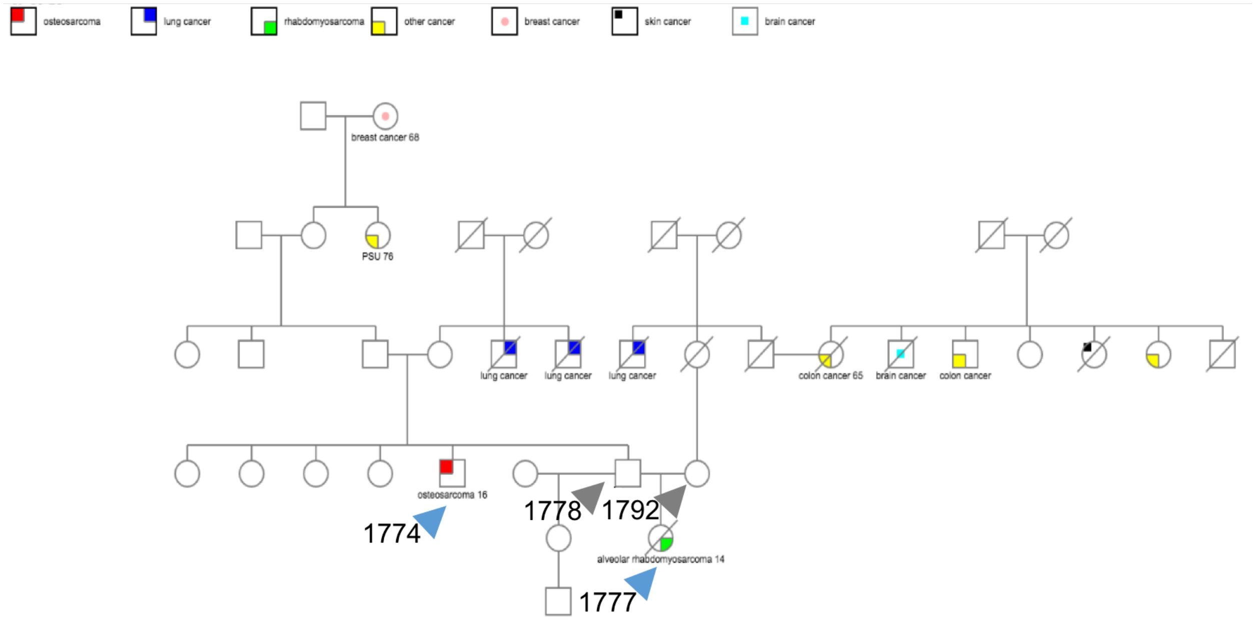

D. WT4 (LFS - Classical)

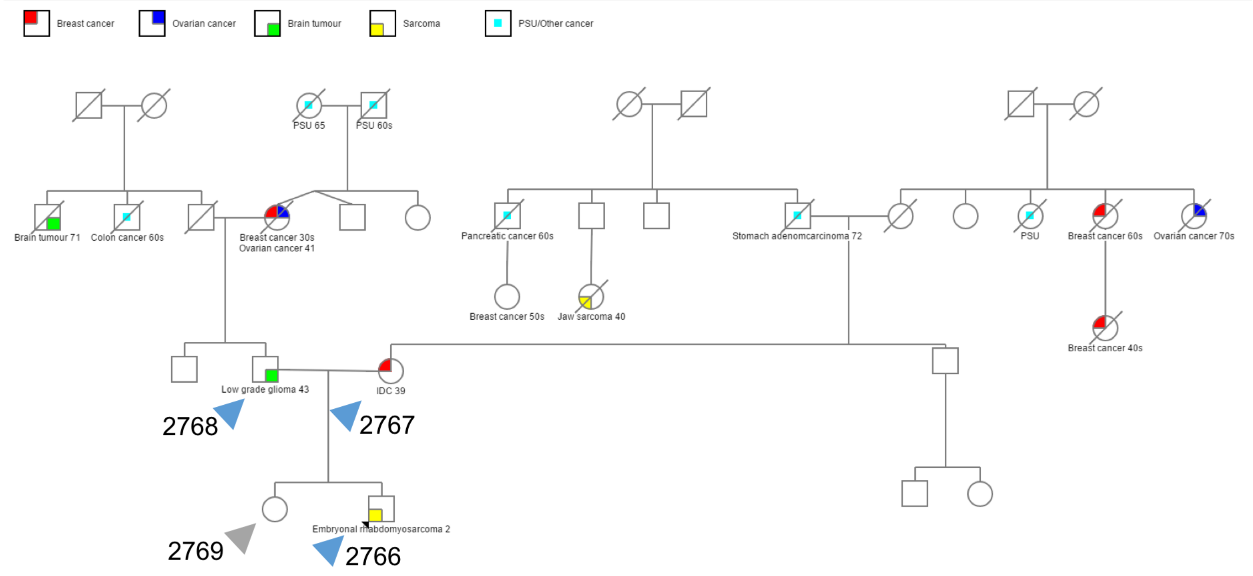

E. WT5 (LFS - Chompret)

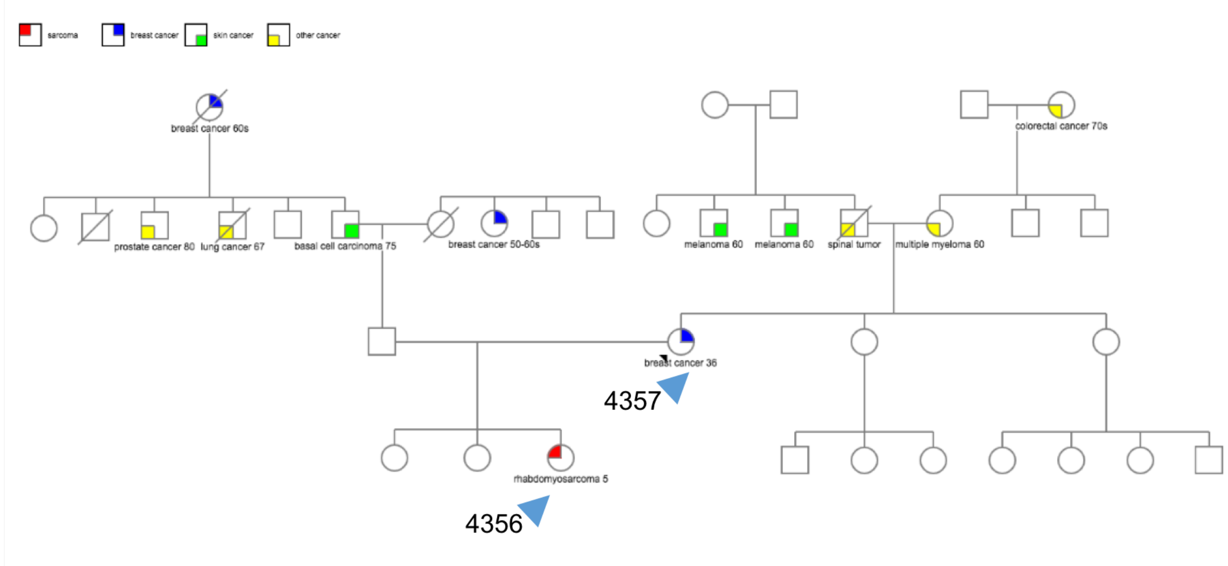

F. WT6 (LFS - Classical)

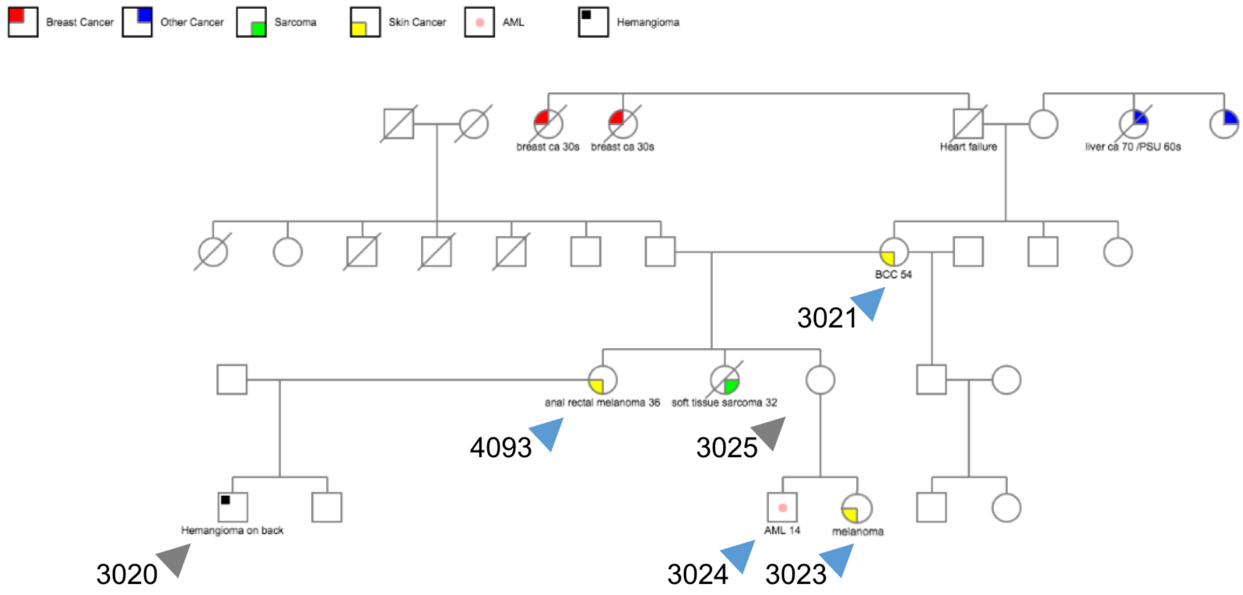

Supplementary Figure S8. Characteristics of significant cis-CSCE. (A) Distribution of distance from probe to associated rsSNP (**B)** Probe distribution in relation to CpG islands (**C)** Probe distribution relative to functional region (**D)** Probe association to cancer status (y-axis) relative to meQTL association (x-axis)

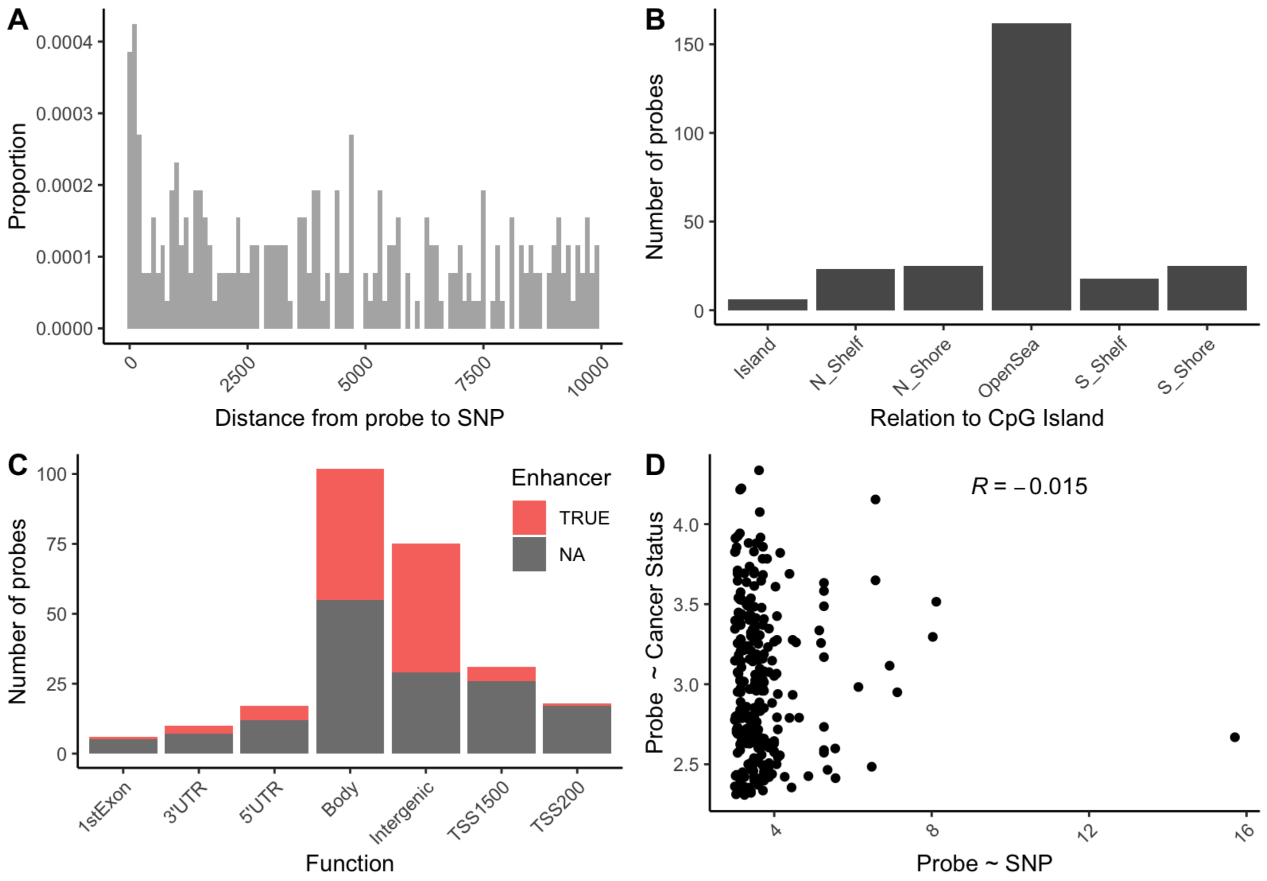

Supplementary Figure S9. UMAP projection of methylation at 259 epimutations coloured by age of cancer onset (grey = unaffected) and tissue type of first cancer.

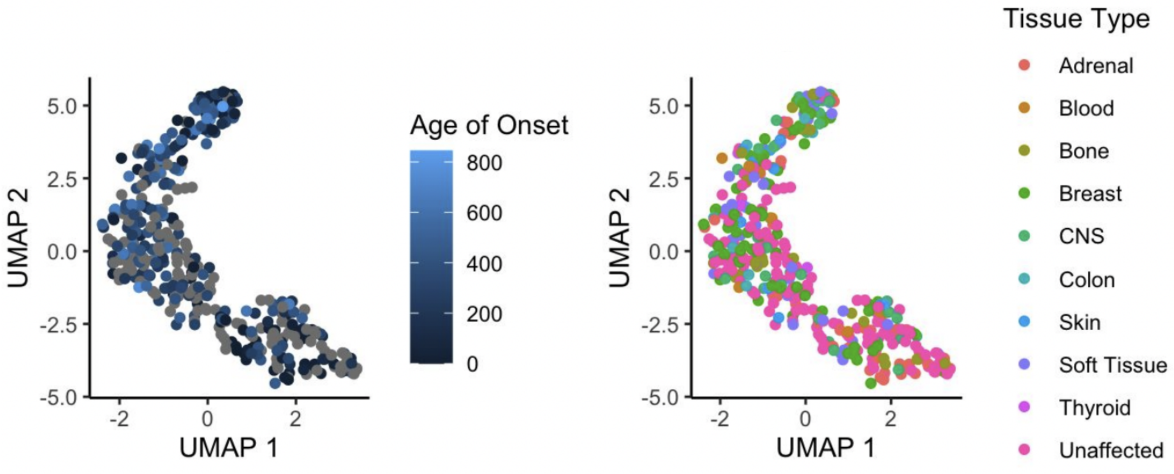

Supplementary Figure S10. Comparison of the methylation of the body of *LEF1* (cg03041109) in **(A)** blood, kidney, nephrogenic rests (NR), Wilms tumours (WT) with favourable histology and WT with anaplastic histology and **(B)** LFS-associated tumours from TCGA. ACC = adrenocortical carcinoma; BRCA = breast cancer; COAD = colorectal cancer; GBM = glioblastoma; LGG = low grade glioma; SARC = sarcoma.

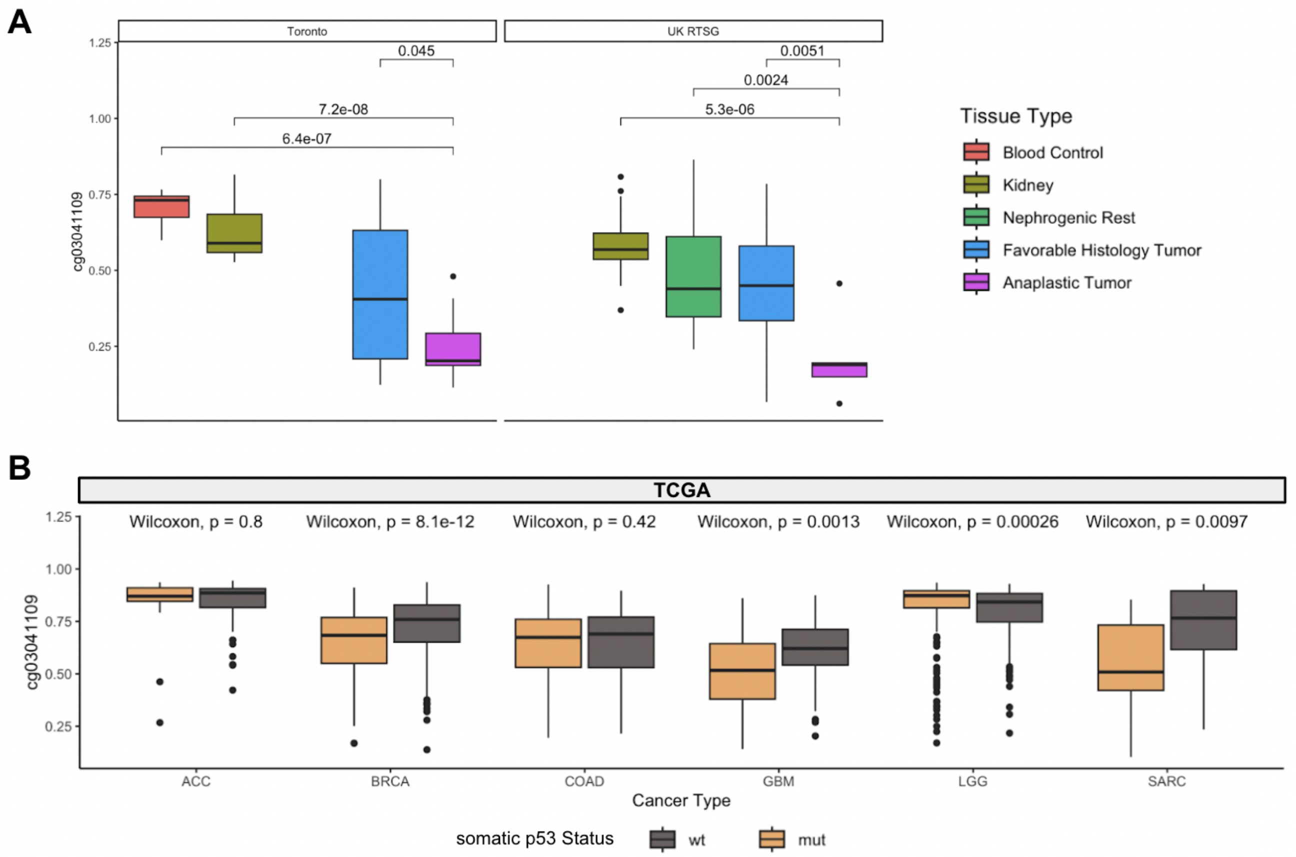

Supplementary Figure S11. Beta density plots before and after dye-bias normalization using ssNoob.

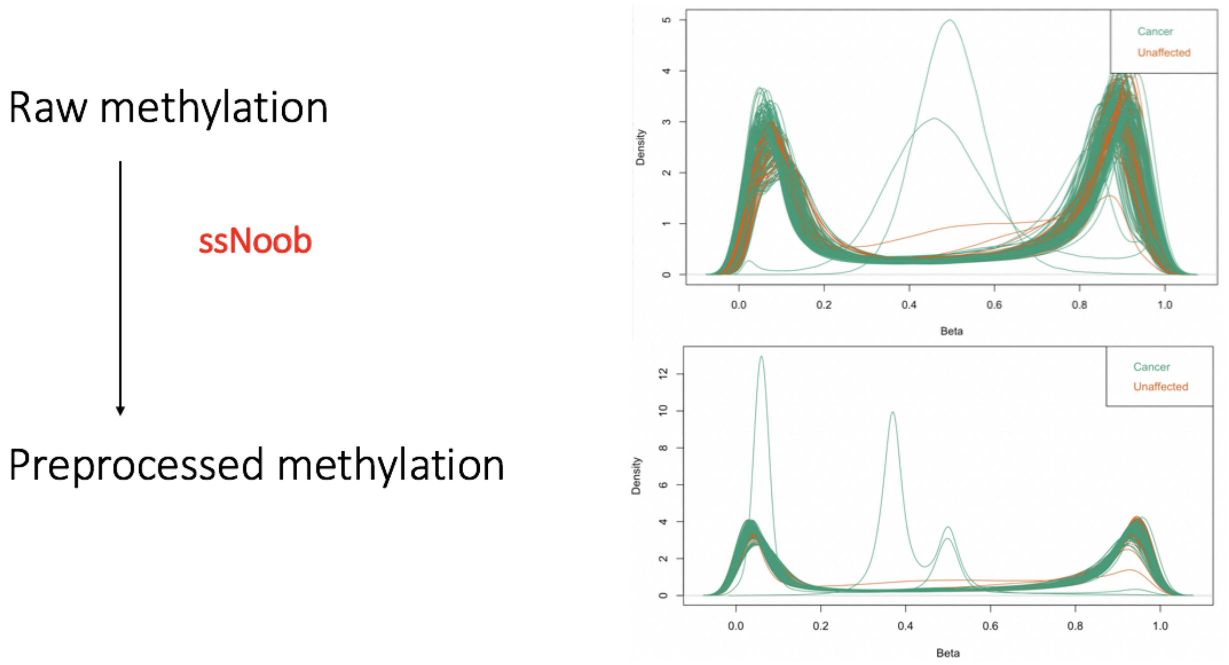

Supplementary Figure S12. PCA before (top) and after (bottom) batch bias correction for discovery (450), validation (850) and external validation (nci) cohorts.

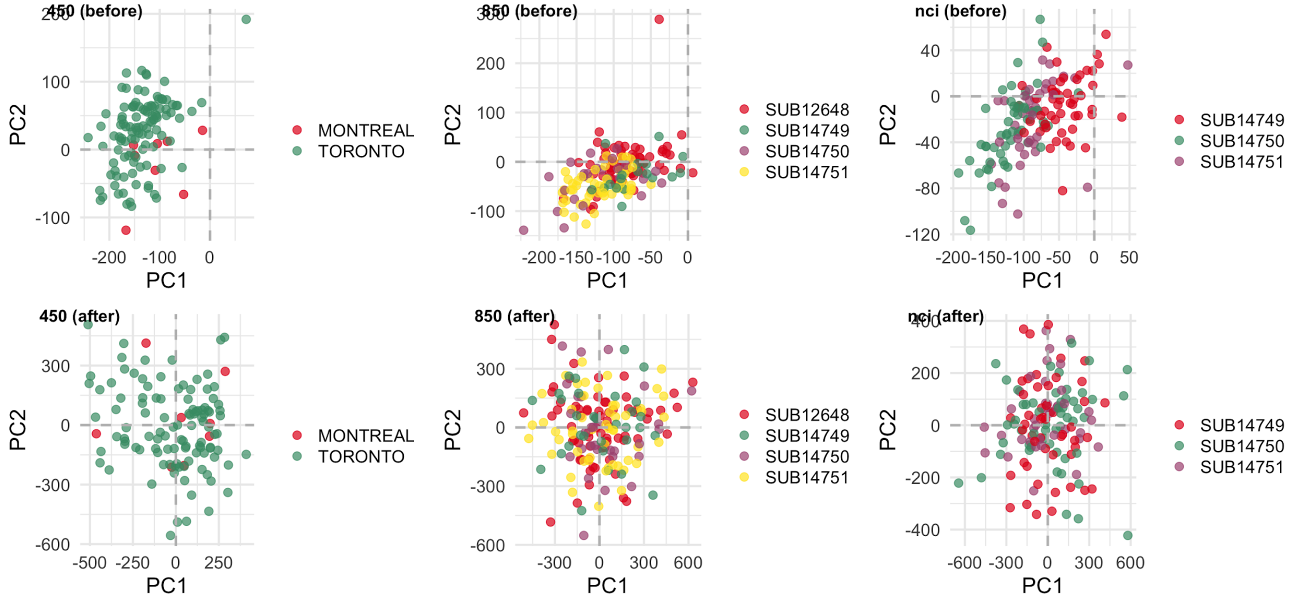

Supplement: Supplementary Figures S1-S12 — Supplementary Figures [file crc-22-0402-s01.pdf]
